# T and NK cell abundance defines two distinct subgroups of renal cell carcinoma

**DOI:** 10.1080/2162402X.2021.1993042

**Published:** 2022-01-04

**Authors:** Moon Hee Lee, Petrus Järvinen, Harry Nísen, Oscar Brück, Mette Ilander, Ilona Uski, Jason Theodoropoulos, Matti Kankainen, Tuomas Mirtti, Satu Mustjoki, Anna Kreutzman

**Affiliations:** a Hematology Research Unit Helsinki, Department of Clinical Chemistry and Hematology, University of Helsinki and Helsinki University Hospital Comprehensive Cancer Center, Helsinki, Finland; b Translational Immunology Research Program, University of Helsinki, Helsinki, Finland; c Abdominal Center, Urology, Helsinki University and Helsinki University Hospital, Helsinki, Finland; d iCAN Digital Precision Cancer Medicine Flagship, Helsinki University Hospital and University of Helsinki, Helsinki, Finland; e Department of Pathology, Helsinki University Hospital and Research Program in Systems Oncology, University of Helsinki, Finland

**Keywords:** RCC, NK cell, T cell, solid tumors, tumor immunology

## Abstract

Renal cell carcinoma (RCC) is considered as an immunogenic cancer. Because not all patients respond to current immunotherapies, we aimed to investigate the immunological heterogeneity of RCC tumors. We analyzedthe immunophenotype of the circulating, tumor, and matching adjacent healthy kidney immune cells from 52 nephrectomy patients with multi-parameter flow cytometry. Additionally, we studied the transcriptomic and mutation profiles of 20 clear cell RCC (ccRCC) tumors with bulk RNA sequencing and a customized pan-cancer gene panel. The tumor samples clustered into two distinct subgroups defined by the abundance of intratumoral CD3+ T cells (CD3^high^, 25/52) and NK cells (NK^high^, 27/52). CD3^high^ tumors had an overall higher frequency of tumor infiltrating lymphocytes and PD-1 expression on the CD8+ T cells compared to NK^high^ tumors. The tumor infiltrating T and NK cells had significantly elevated expression levels of LAG-3, PD-1, and HLA-DR compared to the circulating immune cells. Transcriptomic analysis revealed increased immune signaling (IFN-γ, TNF-α via NF-κB, and T cell receptor signaling) and kidney metabolism pathways in the CD3^high^ subgroup. Genomic analysis confirmed the typical ccRCC mutation profile including *VHL, PBRM1*, and *SETD2* mutations, and revealed *PBRM1* as a uniquely mutated gene in the CD3^high^ subgroup. Approximately half of the RCC tumors have a high infiltration of NK cells associated with a lower number of tumor infiltrating lymphocytes, lower PD-1 expression, a distinct transcriptomic and mutation profile, providing insights to the immunological heterogeneity of RCC which may impact treatment responses to immunological therapies.

## Introduction

Renalcell carcinoma (RCC) is the most prevalent kidney cancer that dominates approximately 90% of all kidney cancers and accounts for one of the top ten most frequent cancer types worldwide.^1,2^ RCC employs multiple different subtypes, of which the clear cell type (ccRCC) is the most common histopathological form.^3,4^ The molecular and genetic characteristics of RCC are distinct for each subtype, having their own histopathological features, clinical outcomes, as well as responses to therapy.^1,4^ Out of the various genetic alterations in ccRCC, the most commonly mutated genes are *VHL, PBRM1*, and *SETD2*.^3,5–9^ As a chemo-resistant cancer, radical nephrectomy remains the treatment of choice in non-metastatic cases and is considered as a potentially curative therapy in localized disease.^10^ However, 20%–30% of patients have a metastasized disease already at the time of diagnosis, and 40% of operated patients develop recurrencies.^10^ First-line treatment options include vascular endothelial growth factor (VEGF)-targeted therapies using tyrosine kinase inhibitors (TKIs) such as pazopanib and sunitinib, as well as immune checkpoint therapy combinations that target PD-1 and CTLA-4.^10,11^ Although current immune checkpoint inhibitor therapies have shown encouraging results in RCC, only a fraction of patients benefit from the treatment, and the majority still have progressive disease.^12^

Immune cells present in the tumor microenvironment (TME) play a crucial role in the progression of cancer and are attractive therapeutic targets. In solid tumors, the highest level of lymphocytic infiltration has previously been observed in ccRCC.^13^ Regarding the immunogenicity of RCC, previous studies have shown that RCC employs a high frequency of T cells,^14,15^ but in contrast to other tumors, CD8+ T cells are not associated with good prognosis.^6,16,17^ Moreover, ccRCC tumors have increased levels of angiogenesis, resulting in a low number of tertiary lymphoid structures and high density of immature dendritic cells that are known to recruit regulatory T cells (T_regs_) associated with the presence of polyclonal, poorly cytotoxic CD8+ T cells.^18,19^ The activation of T cells triggers an upregulation of the inhibitory PD-1 receptor as a normal regulatory step to limit the effector function. However, tumors utilize this pathway as a mechanism for immune evasion by engaging with its major ligand PD-L1, present on tumor and stromal cells, rendering the inhibition of T_effector_ cell functions.^20,21^ LAG-3 is expressed by activated T_regs,_ and binds to the MHC class II HLAs with high affinity, thus augmenting the production of pro-inflammatory cytokines and negatively regulating T cell responses.^20,22,23^ The role of NK cells mediating a strong, antigen independent, anti-tumor immune response^24,25^ is mostly an unexplored realm in RCC, and few studies have associated them with improved survival rates in patients with metastatic disease.^26,27,28^ Because of the counterintuitive role of CD8+ T cells in RCC, NK cell modulating therapies may represent an alternative treatment option.

In this study, we performed a comprehensive immunoprofiling of primary RCC tumor tissue samples and demonstrate that they form two distinct subgroups: NK cell (NK^high^) and T cell (CD3^high^) dominant cases. Our results provide insights into the immunological heterogenicity of RCC tumors and consideration of potential immunological biomarkers in prospective clinical trials.

## Materials and methods

### Patients and study approval

The study included newly diagnosed RCC (n = 45) and non-RCC (n = 7) patients that underwent radical nephrectomies (Table 1). Primary tumor, peripheral blood (PB), and adjacent healthy kidney tissue samples were obtained from the patients during the surgical procedures within a four-year time frame.

**Table 1.** Sample cohort and patient characteristics

Patients	All (n = 52)
**Age in years: mean (range)**	68 (23–85)
**Gender: n (%)**	
Male	29 (55.8)
Female	23 (44.2)
**Histology: n (%)**	
Clear cell	39 (75)
Chromophobe	3 (5.7)
Oncocytoma	5 (9.6)
Papillary	3 (5.7)
Urothelial	1 (1.9)
Angiomyolipoma	1 (1.9)
**WHO/ISUP 2016 tumor grade at diagnosis: n (%)**	
I	1 (1.9)
II	24 (46.1)
III	18 (34.6)
IV	4 (7.7)
Unknown	5 (9.6)
**Deaths due to metastasis: n (%)**	
Yes	3 (5.8)
No	49 (94.2)

The study was approved by the Helsinki University Hospital ethical committee (Dnro 115/13/03/02/15) and was conducted in accordance with the Declaration of Helsinki. All samples were taken after a signed informed consent.

### Sample preparation and processing

Peripheral blood mononuclear cells (PB MNCs) were separated using Ficoll-Paque gradient centrifugation (GE Healthcare, Buckingham, UK), and were stored in cryo vials at −150°C.

Freshly excised tumor samples and their matching adjacent healthy tissue were kept in MACS® tissue storage solution (Miltenyi Biotec 130–100-008) at 4°C upon harvest and were delivered to our facilities within minimal transportation time. Both tissue samples were processed immediately upon delivery and were cut into four fragments: one piece was cut into smaller chunks and frozen as whole fragments in 10% FBS-DMSO freezing medium. Another piece was dissociated using Miltenyi’s Tumor Dissociation kit protocol (Miltenyi Biotec 130–095-929). A third piece was kept in 4% PFA solution in PBS overnight and later processed into formalin-fixed, paraffin embedded (FFPE) blocks. From a proportion of the samples (n = 10), a fourth piece was separately dissociated in order to independently phenotype the spatially different tumor fragments. The freshly dissociated cells were used for flow cytometry analysis using the FACS Verse (BD Biosciences) and the remaining cells were viably frozen and kept at −150°C.

### Tissue preservation and staining

A section of the fresh tumor and matching adjacent healthy kidney tissue samples were cut, processed according to standard FFPE guidelines, and stained with hematoxylin and eosin (HE) onto microscopy slides.

### Multi-parameter flow cytometry and immunophenotyping

Freshly dissociated tumor and adjacent healthy kidney, as well as fresh whole blood samples were used to examine the immune cell numbers and immunophenotypes. The whole blood or dissociated kidney samples were stained for 15 min with a comprehensive antibody staining panel that included markers for immune checkpoint molecules, chemotaxis, cytotoxicity, and cell migration. The full list of markers is found in the supplementary data (Supplementary Table S1). 1 mL of red blood cell lysis buffer was added to each tube and incubated for 10 minutes. The cells were washed twice with PBS before phenotyping. Next, a total of 50 000 lymphocytes were acquired with the FACS Verse (BD Biosciences) and analyzed with FlowJo (Version 10.0.8rl, Treestar). All antibodies were purchased from BD Biosciences (BD Biosciences, San Diego, CA, USA) unless mentioned otherwise.

### Clinical data

We assessed a total of 25 clinical parameters, including diagnostic-phase laboratory values, tumor size and weight, TNM staging, presence of necrosis, perirenal and peripelvic fat infiltration, rhabdoid histology, date of relapse, and other medical histories (Supplementary Table S2).

### Bulk RNA sequencing

From the ccRCC samples, 20 tumors and 10 matching healthy tissues were selected based on the CD3^high^ and NK^high^ abundance according to the heatmap clustering (Supplementary Fig. S1). Details of the RNA isolation, sequencing and standard workflow, data preprocessing and adjustments for possible confounding factors are described in the supplemental methods.

### Pan-cancer gene panel

The selection of the samples (20 tumor samples and their matching adjacent healthy kidney tissue) was chosen based on the same samples that were used for the bulk RNA sequencing. Details of the custom panel sequencing workflow, data preprocessing and variant analysis are described in the supplementary section.

### Statistical analysis

Heatmap clustering was carried out by normalizing with the median scale of the data and using Spearman’s rank correlation as well as ward.D2 linkage methods. Non-parametric Mann-Whitney U-test (unpaired, two-tailed) was used to compare two groups; Kruskal-Wallis test using Dunn’s correction for multiple comparisons with a family-wise alpha threshold and confidence level of .05 was used for three or more groups of continuous variables. Comparisons between the RCC PB and healthy controls were made using the unpaired t test with Welch’s correction. Pearson correlation was used to compare T vs PB, and T vs H tissues. All scatter dot plots show the median and range as horizontal lines. The statistical analyses were computed using Prism 8 (GraphPad Software Inc.) and RStudio version 4.0.2. For all graphs: ns, not significant, *p < .05; **p < .01; ***p < .001; ****p < .0001.

### Multiplexed immunohistochemistry (mIHC) and imaging of whole tissue slides

FFPE whole tissue from 11 tumors and 11 matching adjacent healthy renal tissue were cut into 4 mm thin slides and stained for CD4+ and CD8+ T cells. Briefly, the FFPE blocks were deparaffinized and stained with the following antibodies: (panel 1) FOXP3, CD3, CD4, CD8, (panel 2) CD45, Carbonic Anhydrase IX (CAIX), E-cadherin and pan-cytokeratin. Further technical details are found in previous publications^29–31^and the supplemental methods. The quality of the scanned and processed images was visually assessed.

To reduce autofluorescence, we merged multiple channels (GFP, Cy3, Cy5) together. Then, we trained a pixel classifier with the software Ilastik 1.3.2 to detect autofluorescence with the following features: color/intensity (sigma .3, 1.0, 3.5, 10.0), edge (sigma .7, 1.6, 5.0) and texture (sigma .3, 1.0, 3.5, 10.0). We exported the detected autofluorescence mask as a binary image. Then, we calculated the proportion of immune cells with pixel calculation using Fiji 2.0.0.^31^ First, areas corresponding to the autofluorescence mask were excluded. Then, images were converted to binary format with the default thresholding method based on the IsoData algorithm.^32^ The area of marker-positive cells (CD3, CD4, CD8) was calculated from the thresholded images and compared to the area of DAPI-positive cells.

## Results

### RCC tumors are divided into two subgroups based on the prevalence of T and NK cells

**Figure 1.**
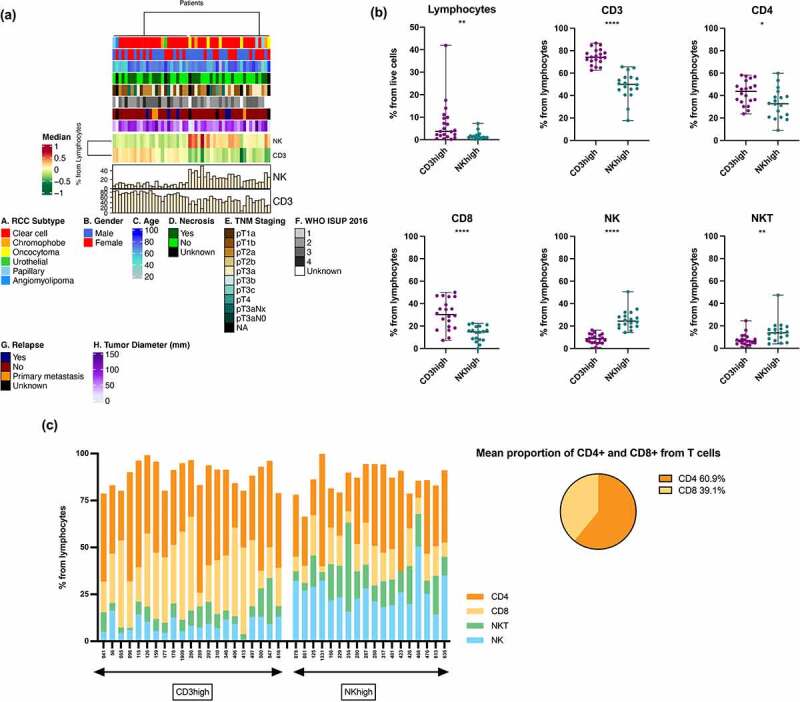
Heatmap of the RCC cohort and analysis of the lymphocyte subsets according to the CD3^high^ and NK^high^ subgroup clusters. (a) Heatmap showing RCC cohort (all subtypes included) according to the intratumoral CD3+ T cell and NK cell abundance using Spearman correlation and ward.D2 clustering methods. The bottom histograms show the percentage of CD3+ T and NK cells from the lymphocyte population. Patient characteristics including gender, age, presence of necrosis, TNM staging, WHO/ISUP 2016 tumor grading, relapse state and tumor sample diameter are shown as color keys above the heatmap. Benign oncocytoma cases are clustered to the NK^high^ subgroup. A heatmap showing only the ccRCC cases is found in Supplementary Fig. S1A. (b) Statistical analysis (Mann-Whitney U test) comparing the intratumoral CD3^high^ and NK^high^ subgroups according to the abundance of the lymphocyte subsets. The CD3^high^ subgroup encompassed a larger percentage of lymphocytes, CD3+, CD4+, and CD8+ T cells compared to the NK^high^ subgroup. The NK^high^ subgroup constituted an increased abundance of NK and NKT cells. ns, not significant, *p < .05; **p < .01; ***p < .001; ****p < .0001. (c) Proportion of CD4+, CD8+ T cells, NK and NKT cells from the intratumoral lymphocyte population. The x-axis shows patients categorized into the CD3^high^ and NK^high^ subgroups. The pie chart represents the mean proportion of CD4+ and CD8+ T cells from both subgroups.

To first assess the immunological landscape of all the tumors, we performed extensive flow cytometric immunophenotyping of the freshly homogenized tumor samples. In total, our cohort included 52 cases out of which 39 (75%) were ccRCC (Table 1). Five patients (9.6%) were benign oncocytoma cases confirmed by histopathological analysis; the rest of the cases were diagnosed with chromophobe, papillary, urothelial or angiomyolipoma histologies. The main lymphocyte subpopulations (T and NK cells) were gated based on the CD45+ lymphocytes on CD3 and CD56 expressions, and heatmap analysis was carried out based on the frequency of the cells in the tumor. We observed that the tumors divided into two distinct clusters based on their T and NK cell abundance and were classified as CD3^high^ and NK^high^ subgroups, respectively (Figure 1a). The cutoffs for the subgroups were based on the Spearman’s correlation distance and ward.D2 hierarchical clustering methods. Similar clustering to NK^high^ and CD3^high^ phenotypes were also noted when only the patients with the most common ccRCC subtype were included (Supplementary Fig. S1A). Additionally, to observe the spatial distribution and infiltration of the T cells, we carried out mIHC from 11 whole tissue slide samples (CD3^high^ (n = 5) and NK^high^ (n = 6) subgroups) (Supplementary Fig. S2A-C). Although no statistical differences were observed, increased median levels of expressions for the CD3+, CD4+, CD8+ and FOXP3+ markers were detected in the CD3^high^ group compared to the NK^high^ subgroup, in line with the results from our flow cytometry heatmap analysis (Supplementary Fig. S1C).

From the heatmap, we also noticed that the benign oncocytoma cases (n = 5) clustered to the NK^high^ subgroup (Figure 1a). When the differences in the immune cell subtypes between the benign oncocytoma and malignant RCC (clear cell, chromophobe, papillary) cases (n = 45) were analyzed, we found that the oncocytoma cases had a lower percentage of CD3+ (*p* = .0082 median 34% vs 64.5%) and CD4+ (*p* = .038 median 23.3% vs 36.4%) T cells. Oncocytomas seem to have a higher percentage of NK cells than the malignant tumors, but due to small number of cases, no statistical significance was reached (*p* = .17 median 25% vs 14.1%) (Supplementary Fig. S1B).

To guarantee a more homogenous patient population and consistency of results, only ccRCC patients were included in the follow-up analyses. Comparing the total lymphocyte infiltration in the tumors, we noted that the CD3^high^ group had a significantly higher percentage of lymphocytes (*p* = .0096, median 3.7% vs 1.2%) (Figure 1b). Gating strategies of two representative cases (lymphocyte-rich and -poor), as well as marker expressions for each immune cell subset are found in the Supplementary Fig. S3A-E. In the NK^high^ group, all patients had less than 10% of tumor infiltrating lymphocytes, and the median percentage of NK cells in this subgroup was 24.4% compared to 8.7% in the CD3^high^ group (Figure 1b). In one ccRCC case, the NK cell percentage was notably high, 50.5% (Figure 1b,c). The proportion of CD3+ T cells from the lymphocytes was higher in the CD3^high^ group (*p* < .0001, median 74.1% vs 50%) as expected, but T cells were also present in the NK^high^ group, constituting approximately 50% of the lymphocytes. The proportion of CD4+ (*p* = .02 median 43.7% vs 32.7%) and CD8+ (*p* < .0001 median 30.2% vs 14.8%) T cells was higher in the CD3^high^ than in the NK^high^ subgroup, respectively. Additionally, the proportion of NKT cells was higher in the NK^high^ group (*p* = .0033, median 13.7% vs 6.4%) (Figure 1b). The proportion of CD4+ and CD8+ T cells from both subgroups combined was 60.9% and 39.1%, respectively (Figure 1c).

Clinical parameters such as gender, presence of necrosis, TNM staging, WHO ISUP 2016 tumor grade, relapse status and tumor diameter (Supplementary Table S2) did not significantly differ between the subgroups (Figure 1a, Supplementary Fig. S4A, B). However, ccRCC patients in the NK^high^ group were slightly older than patients in the CD3^high^ group (*p* = .028, median years 73.5% vs 70%) (Supplementary Fig. S4A).

### NK cell dominant tumors have a unique immune profile

To further study the differences between the CD3^high^ and NK^high^ tumors, we analyzed different immunological markers with an extensive panel that included activation, immune checkpoint, migration, and memory markers: CX3CR1, CD16, CD3, CD4, TCR GammaDelta, CD45, CD8, CD56, PD-1, LAG-3, ICOS, CTLA-4, HLA-DR, CD27, CD25, CD11b, NKG2C, CD161, NKG2D, NKG2A, DNAM, CD57, NKp46, NKp30, CXCR3, CCR-7, CD45RO, CXCR4, PD-L1, and PD-L2. Full details of the panel are found in the supplementary material (Supplementary Table S1).

**Figure 2.**
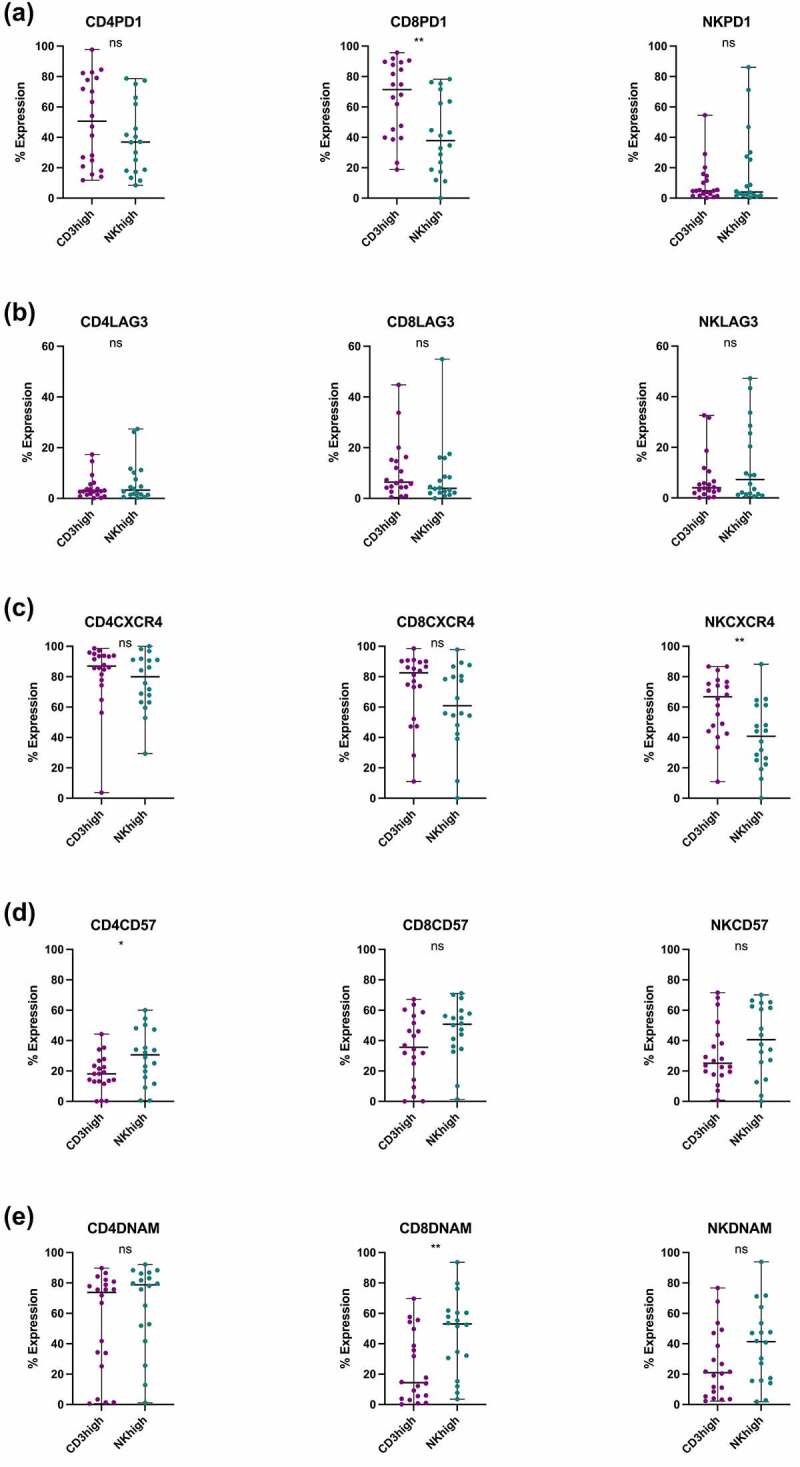
Comparison of marker expressions between the CD3^high^ and NK^high^ subgroups. (a) The proportion of PD-1 positive CD4+, CD8+ T and NK cells in the subgroups. (b) The proportion of LAG-3 positive CD4+, CD8+ T and NK cells in the subgroups. (c) The proportion of CXCR4 positive CD4+, CD8+ T and NK cells in the subgroups. (d) The proportion of CD57 positive CD4+, CD8+ T and NK cells in the subgroups. (e) The proportion of DNAM positive CD4+, CD8+ T and NK cells in the subgroups. (a)-(e). All statistical analyses were done using Mann-Whitney U test: ns, not significant, **p < .01.

Higher PD-1 expression was observed in the CD3^high^ CD8+ T cells compared to the NK^high^ group (*p* = .0049, median 71.4% vs 37.9%), whereas no differences were identified for the expression of PD-1 in CD4+ T and NK cells between both subgroups (Figure 2a). In contrast, LAG-3 expressions remained similar across all the studied lymphocytes (CD4+, CD8+ T and NK cells) in the CD3^high^ and NK^high^ groups (Figure 2b). Elevated CXCR4+ expression in the NK cells was observed in CD3^high^ tumors compared to the NK cell rich tumors (*p* = .0049, median 66.8% vs 40.8%) (Figure 2c). The NK^high^ tumors also showed higher prevalence of CD57 expressing CD4+ T cells (*p* = .0450, median 30.6% vs 18.2%) (Figure 2d), as well as DNAM+ CD8+ T cells (*p* = .0084, median 53.0% vs 14.4%) (Figure 2e). No differences in expressions were observed for all other markers (Figure 2b-e).

### Comparison between tumor and matching adjacent healthy kidney tissue reveals immunological differences

**Figure 3.**
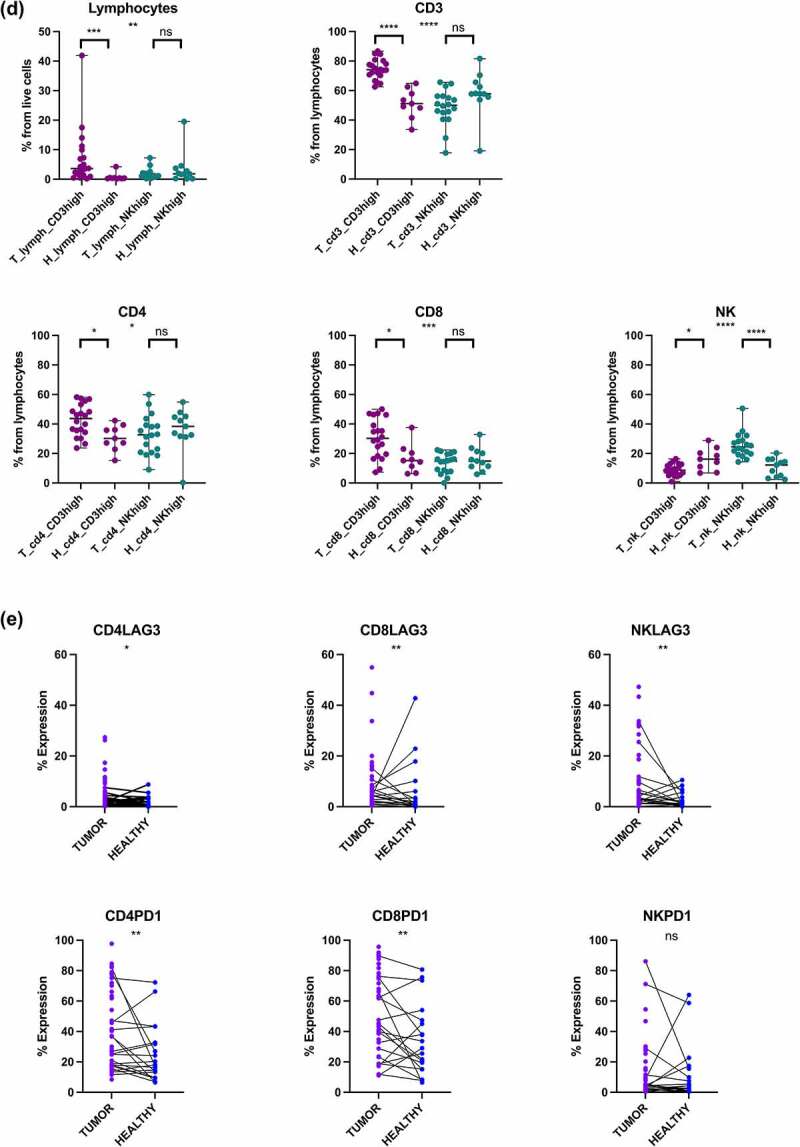
(Continued).

Initially, patient tumor (T) and matching adjacent healthy renal tissue (H) samples were collected upon surgical nephrectomy, histologically confirmed by the corresponding pathologist, fixed and HE stained. Two representative cases (Figure 3a) show varying degrees of cancerous tumor cells (40%-90%) occupying the tumor tissue, as well as the regions of lymphocyte-poor or -rich infiltration respectively, whereas the matching healthy tissue histologically mirror the normal cortical tissue of the kidney.
**Figure 3.**
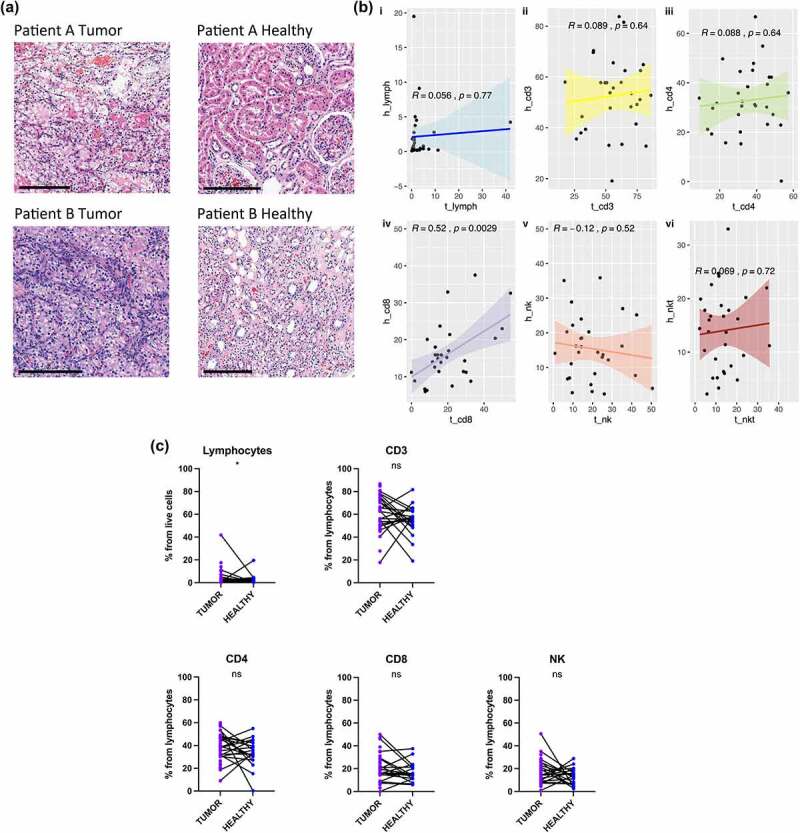
Immunological changes between the tumor and matching adjacent healthy tissue. (a) Examples of renal tissue histology in the hematoxylin and eosin (HE) stained microscopy slides. Varying degrees of cancerous tumor cells (Patient A 40% and Patient B Tumor 100%) occupying the tumor tissue, as well as the regions of lymphocyte-poor or -rich infiltration respectively. The matching adjacent healthy kidney tissue mirror the normal cortical tissue of the kidney (Patient A and Patient B Healthy). (b) Comparison between the different immune subsets (CD3+, CD4+, CD8+ T cells, and NK cells) between the tumor (T) and adjacent healthy (H) kidney tissue. Statistical analysis was performed using Kruskal-Wallis non-parametric test: ns, not significant; *p< .05; **p < .01; ***p < .001; ****p < .0001. (c) The proportion of different lymphocyte subsets in the paired tumor (T) and healthy (H) tissue samples. Mann-Whitney U test: ns, not significant; *p< .05. (d) Comparison of the different immune subsets (CD3+, CD4+, CD8+ T and NK cells) between the tumor (T) and adjacent healthy (H) kidney tissue in the CD3^high^ and NK^high^ subgroups. Kruskal-Wallis test: ns, not significant; *p < .05; **p < .01; ***p < .001; ****p < .0001. (e) The proportion of LAG-3 positive CD4+, CD8+ T and NK cells between the tumor and healthy tissue samples. Mann-Whitney U test: *p < .05; **p < .01. (f) The proportion of PD-1 positive CD4+, CD8+ T and NK cells between the tumor and healthy tissue samples. Mann-Whitney U test: ns, not significant; **p < .01.

Next, we compared different immune subsets (CD3, CD4+, CD8+ T cells, and NK cells) between the tumor and adjacent healthy kidney tissue (Figure 3b, Supplementary Fig. S5). From the various lymphocyte subclasses, only the CD8+ T cell abundance correlated relatively well between the matched tissue samples (R = .49, *p* = .026; CD3^high^ subgroup R = .67, *p* = .05) (Figure 3biv and Supplementary Fig S5Avii, respectively). Overall, tumors contained a greater proportion of lymphocytes compared to the matching healthy tissue (*p* = .021, median 1.9% vs .5%) (Figure 3c). When the subgroups were analyzed separately, the higher lymphocyte proportion was only observed in the CD3^high^ subgroup (*p* = .0003, median 3.6% vs .3%) (Figure 3d). In the CD3^high^ subgroup, the total proportion of tumor infiltrating lymphocytes also correlated well between the tumor and healthy tissues (R = .96, *p* < .0001) (Supplementary Fig S5Ai). The proportion of CD3+ T cells was also higher in the tumor tissue for the CD3^high^ group when compared to the adjacent healthy tissue (*p* < .0001, median 74.0% vs 51.1%), and a similar observation was made for NK cells in the NK^high^ group (*p* < .0001, median 24.4% vs 12.2%) (Figure 3d). The CD3^high^ subgroup also had increases in the CD4+ (*p* = .026, median 43.7% vs 30.2%) and CD8+ T cells (*p* = .017, median 30.3% vs 15.2%) compared to their healthy tissue counterparts (Figure 3d).

When further comparing the clinically relevant markers LAG-3 and PD-1, the intratumoral immune cells in the CD3^high^ subgroup showed a higher expression of CD4+ LAG-3 (*p* = .01, median 2.8% vs .2%), CD8+ LAG-3 (*p* = .02 median 6.4% vs .9%), CD4+ PD-1 (*p* = .02, median 50.6% vs 18.1%) and CD8+ PD-1 (*p* = .008, median 71.4% vs 25.5%), whereas no difference was found in the NK^high^ group between the tumor and the healthy tissue (Figure 3e,f and Supplementary Fig. S5B, respectively).

The tumor infiltrating CD4+ and CD8+ T cells also had a mature immunophenotype, as they were more often CD57 positive and CD27 negative, as well as expressing the chemokine receptor, CXCR4 (Supplementary Fig. S6A-C). The only difference observed in the NK cells was the activating receptor NKG2D+ expression, which was higher in the tumor compared to the matching healthy tissue (*p* = .0029, median 31.2% vs 17.3%) (Supplementary Fig. S6D).

In order to investigate the spatial heterogeneity of the tumors, we independently phenotyped two alternate regions (t1, t2) of the tumor sample (Supplementary Fig S7). We observed strong correlations between the immune cell abundancies of the distinct regions: lymphocytes R = .88 (*p* = 6.2x10^−5^), CD3+ R = .84 (*p* = .00018), CD4+ R = .92 (*p* = 2.8x10^−6^), CD8+ R = .57 (*p* = .032), NK R = .55 (*p* = .043), and NKT cells R = .55 (*p* = .043) (Supplementary Fig. S7A-F).

### Circulating lymphocyte profiles differ from tumor phenotypes

**Figure 4.**
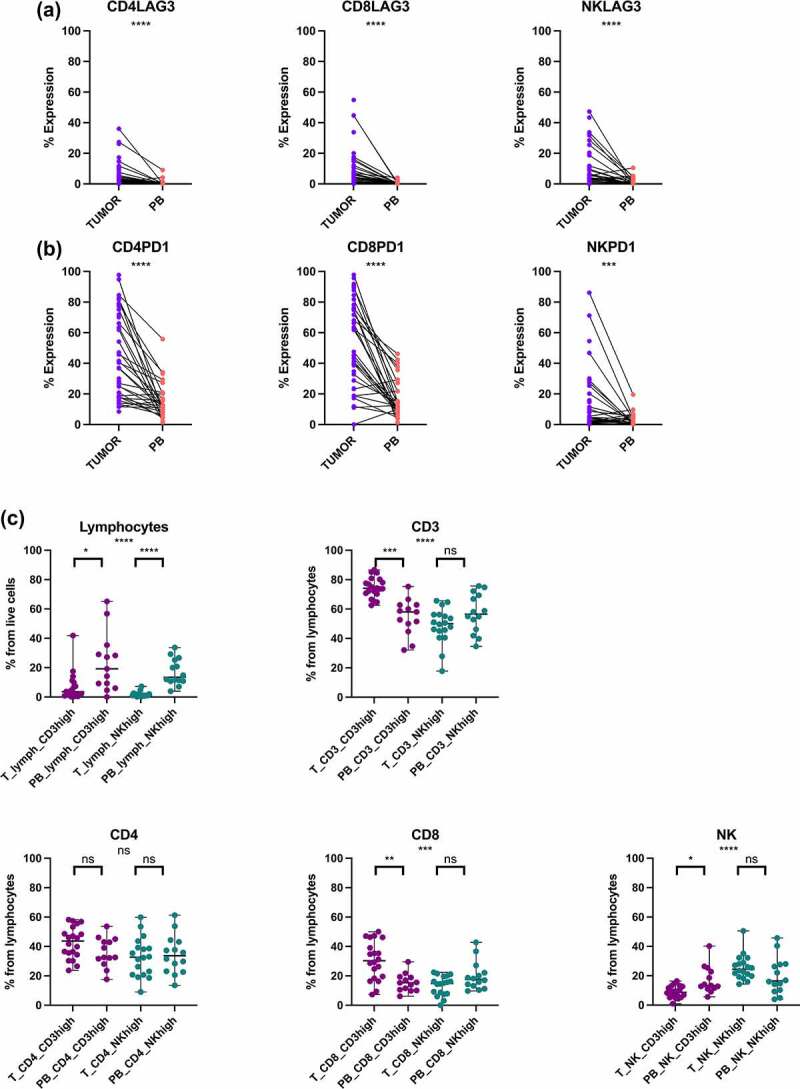
Differences in the circulating and tumor lymphocyte phenotypes. (a) The proportion of LAG-3 positive CD4+ and CD8+ T and NK cells between tumor and peripheral blood (PB) samples. Mann-Whitney U test: ****p < .0001. (b) The proportion of PD-1 positive CD4+ and CD8+ T and NK cells between tumor and peripheral blood (PB) samples. Mann-Whitney U test: ***p < .001; ****p < .0001. (c) The proportion of lymphocytes from live cells, CD3+, CD4+, CD8+ T cells, and NK cells from lymphocytes in the tumor (T) and peripheral blood (PB) samples between the CD3^high^ and NK^high^ subgroups. Kruskal-Wallis test: ns, not significant; *p < .05; **p < .01; ***p < .001; ****p < .0001.

Similar to the tumor and healthy tissue comparisons, the immune subsets were initially compared between the matching tumor (T) and peripheral blood (PB) samples (Supplementary Fig. S8A, B). LAG-3 and PD-1 were expressed more in the tumor infiltrating than circulating immune cells: LAG-3+ CD4+ (*p*< .0001, median 2.80% vs .21%; CD3^high^ *p* = .0001, median 2.8% vs .2%; NK^high^ *p* = .0005, median 3.3% vs .4%), LAG-3+ CD8+ T (*p* < .0001, median 5.26% vs .09%; CD3^high^ *p* < .0001, median 6.4% vs .1%; NK^high^ *p* = .0004, median 4.0% vs .1%), LAG-3+ NK cells (*p* < .0001, median 4.77% vs .62%; CD3^high^ *p* = .02, median 4.0% vs .9%; NK^high^ *p* = .0019, median 7.3% vs .6%), PD-1+ CD4+ (*p* < .0001, median 41.40% vs 11.50%; CD3^high^ *p* < .0001, median 50.6% vs 11.5%; NK^high^ *p* = .01, median 36.8% vs 11.7%), PD-1+ CD8+ T (*p* < .0001, median 62.1% vs 12.4%; CD3^high^ *p* < .0001, median 71.4% vs 12.4%; NK^high^ *p* = .03, median 37.9% vs 12.1%) and PD-1+ NK cells (*p* = .0006, median 4.70% vs 1.52%; NK^high^ *p* = .02, median 4.1% vs 1.1%) (Figure 4a,b, Supplementary Fig. S8C, D).

We next explored other marker expressions between the tumor-PB pairs (Table 2) and found higher CD57+ expression in the tumor CD4+ T cells, whereas in the NK cells, higher expression was observed in the PB samples. Consequentially, DNAM+ expression levels were lower in the intratumoral NK cells, CD4+, and CD8+ T cells, suggesting a less functional phenotype of T and NK cells in the tumor microenvironment, as DNAM expression is related to better IFN-γ production.^33^ Also, CX3CR1+ expression levels in the CD8+ T cells and NK cells were higher in the PB samples compared to the tumor cells. The expression levels of chemokine receptor CXCR3 typically expressed in activated T and NK cells were also higher in the PB CD4+, and CD8+ T cells, but no differences were observed in the NK cell cells. Similarly, CD25+ levels were higher in the PB CD4+ and CD8+ T cells. Elevated CD45RO+ expression in the intratumoral CD4+, CD8+ T and NK cells, and lower CD27+ expression in the same cell subtypes were observed; suggesting again a more mature immune cell phenotype in the tumors compared to the PB samples. In addition, increased HLA-DR+ expression in the tumoral CD4+, CD8+ T and NK cells was found compared to the PB samples. A complete list of the median, range and *p*-values are found in Table 2.

**Table 2.** Changes in the lymphocyte subsets and marker expressions between the CD3^high^ and NK^high^ tumor-PB pairs

		Median		Range		*p* (Mann-Whitney)
	Subsets	T	PB	T	PB	T vs PB
	Lymphocytes	1.90	15.80	0.17–41.90	0.06–65.10	<.0001
	CD3+	64.70	58.00	17.80–86.80	32.10–75.70	.24
	CD4+	37.30	32.50	9.08–59.90	13.50–61-30	>.99
	CD8+	19.50	15.90	.00–50.00	6.12–42.80	.53
	NK	14.50	14.70	.94–50.50	3.92–45.70	>.99
	NKT	8.51	9.40	1.05–47.40	3.16–25-70	>.99
Markers						
CD57	CD4CD57	22.45	6.10	.00–99.20	.00–27.70	<.0001
	CD8CD57	43.55	34.40	.00–78.00	.00–69.40	.15
	NKCD57	26.90	49.85	.10–71.50	.17–88.40	.0050
DNAM	CD4DNAM	75.40	84.95	.64–92.10	28.80–95.70	.0075
	CD8DNAM	48.10	75.10	.22–98.40	13.00–96.60	<.0001
	NKDNAM	30.15	80.30	.35–93.90	3.57–97.90	<.0001
CX3CR1	CD4CX3CR1	5.69	4.99	.11–29.00	.29–49.10	>.99
	CD8CX3CR1	7.63	44.85	.83–21.60	5.44–81.70	.0001
	NKCX3CR1	37.10	93.95	4.91–88.80	38.60–100.00	<.0001
CXCR3	CD4CXCR3	5.55	35.65	.00–94.70	.72–63.70	.0001
	CD8CXCR3	7.44	58.00	.00–99.20	.00–93.80	<.0001
	NKCXCR3	9.90	10.50	.00–97.40	1.06–38.50	>.99
CD25	CD4CD25	3.13	43.85	1.01–11.50	9.20–99.30	<.0001
	CD8CD25	1.63	10.55	.17–5.99	.65–100.00	.0001
	NKCD25	4.70	1.39	.21–15.00	.22–66.40	.43
CD45RO	CD4CD45RO	94.35	80.25	.07–99.30	35.80–97.80	.0018
	CD8CD45RO	85.15	64.60	.88–98.70	23.00–97.10	.015
	NKCD45RO	24.80	5.84	.22–53.30	.50–34.10	.0006
CD27	CD4CD27	3.31	89.05	.10–17.90	56.00–97.00	<.0001
	CD8CD27	4.50	62.10	.28–20.90	34.80–96.00	<.0001
	NKCD27	1.51	4.92	.26–6.11	.78–18.20	.0003
HLA-DR	CD4HLADR	55.00	9.27	24.00–91.00	2.62–22.80	<.0001
	CD8HLADR	59.15	16.45	42.70–96.20	1.95–79.50	<.0001
	NKHLADR	48.65	6.90	12.80–86.50	1.77–25.50	<.0001

Percentage expression of each marker (%) for each immune cell subset and relevant markers showing the median and range percentage values. All median and range values for the immune subset population (CD3, CD4, CD8, NK, NKT) other than the lymphocytes (from total “live cell” population) have been calculated from the lymphocyte population. T = tumor, PB = peripheral blood, H = adjacent healthy renal tissue.

We further compared the immune cell subsets from the tumor and the matching PB samples in the CD3^high^ and NK^high^ subgroups separately. An increase in the circulating PB lymphocytes was found for both CD3^high^ (*p* = .012, median 19.2% vs 3.6%) and NK^high^ (*p* < .0001, median 13.4% vs 1.1%) subgroups, whereas the proportion of intratumoral CD3+ (*p* = .0002, median 74.1% vs 58.0%) and CD8+ (*p* = .002, median 30.2% vs 15.4%) T cells was higher in the CD3^high^ and not in the NK^high^ subgroup (Figure 4c). A full list of the different immune cell subsets as well as marker expressions between the tumor, PB and matching healthy tissues are found in the Supplementary Table S3.

### Transcriptional profiling reveals differences in tumor tissue profiles and gene expression signatures

In order to gain further insights on our findings of the distinct tumor subgroups, we performed bulk RNA sequencing from 11 CD3^high^ and 6 NK^high^ ccRCC cases with the availability of sample material. First, using principal component analysis (PCA), we found that CD3^high^ and NK^high^ tumors did not cluster apart from each other based on the full transcriptomic profile (Figure 5a). Next, we analyzed the differentially expressed genes (DEGs) between the CD3^high^ and NK^high^ subgroups. After adjusting for possible confounding factors, we identified 314 DEGs between the CD3^high^ and NK^high^ subgroups (nominal *p* < .05, absolute logFC > 2) (Figure 5b). Among the most upregulated genes in the CD3^high^ subgroup were the solute carrier genes (*SLC5A12, SLC22A7*) belonging to the family of renal-specific transporter proteins involved in metabolic pathways, genes involved in fatty acid metabolic pathways (*HMGCS2, PLA2G12B*) and a transcriptional target of p53 (*PRAP1*) (Figure 5b,c). Interferon and inflammatory-related genes belonging to the complement pathway (*LY6E, HHLA2, CFB, C6* and *CCL20*) were also increased in the CD3^high^ subgroup (Figure 5b). The CD3^high^ subgroup was also associated with a decreased expression of *CD274* (PD-L1), the ligand that binds with the PD-1 receptor and acts to block T cell activation. Chemokine receptor genes related to immune surveillance (*CXCR12, ACKR2*), antigen recognition (*IGLV1-51*), MHC I antigen processing and presentation (*CD300LG*) were overexpressed in the NK^high^ compared to the CD3^high^ subgroup (Figure 5b). We further carried out heatmap analysis of the six significant DEGs (*p*-adjusted < .05, absolute logFC > 2) together with the corresponding clinical parameters, which confirmed the upregulation of *SLC5A12* and *HMGCS2* in the CD3^high^ subgroup (Figure 5c). Next, gene set enrichment analysis (GSEA) was carried out using the MSigDB 2020 Hallmark and Biocarta 2016 gene sets as references known to encompass various biological and biochemical pathways. Our GSEA analysis further confirmed the findings, and the IFN-γ, TNF-α, MAPK, as well as T cell receptor signaling pathways were among the top ten upregulated pathways in the CD3^high^ subgroup (Figure 5d,e), whereas the Wnt-β catenin, IL-6 mediated JAK-STAT3, PI3K-AKT/mTOR, caspase and NF-κB signaling pathways were among the most downregulated pathways (Supplementary Tables S4, S5).

**Figure 5.**
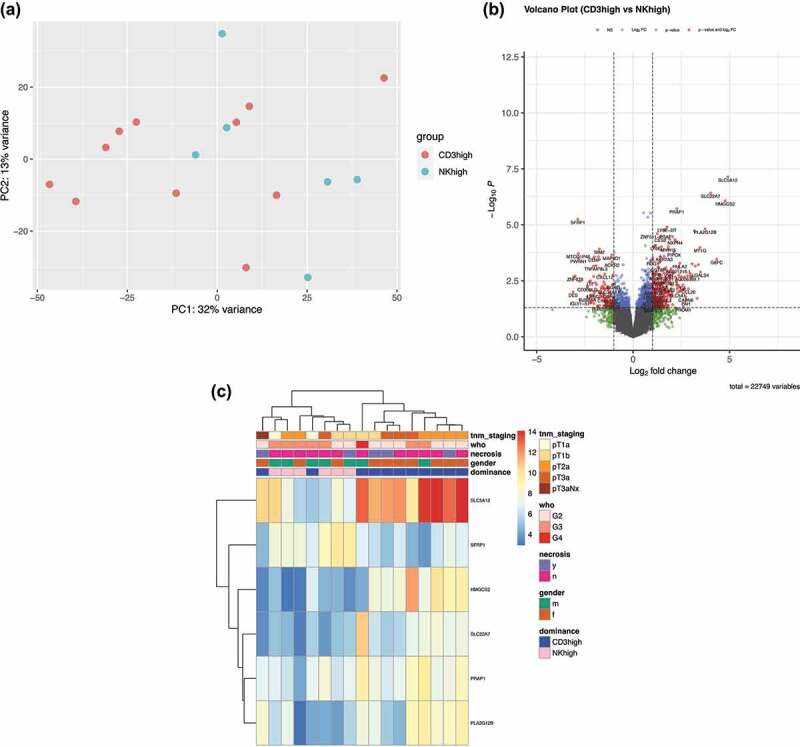
Bulk RNA sequencing reveals transcriptional differences and gene expression signatures in the tumor tissue. (a) Principal component analysis (PCA) from 11 CD3^high^ and 6 NK^high^ ccRCC cases. CD3^high^ and NK^high^ tumors did not cluster apart from each other based on the full transcriptomic profile. (b) Differential gene expression (DEG) analysis between CD3^high^ and NK^high^ subgroups (nominal *p* < .05, absolute logFC > 2). (c) Heatmap showing significant DEGs (*p*-adjusted < .05, absolute logFC > 2) and clinical parameters in ccRCC tumors. The fading blue color indicates downregulation of the gene and red indicates upregulation relative to the mean expression of the genes across all samples. Clinical annotations have been added as colored keys at the top of the heatmap; tnm_staging = TNM classification of malignant tumors, who = WHO ISUP 2016 tumor grading, necrosis = presence of necrosis (y = yes, n = no), gender, dominance = CD3^high^ and NK^high^ subgroups. (d and e) Gene set enrichment analysis (GSEA) using the MSigDB 2020 Hallmark and Biocarta 2016 signatures, showing IFN-γ, TNF-α, MAPK and T cell receptor signaling pathways among the top ten upregulated pathways in the CD3^high^ subgroup. Wnt-β catenin, IL-6 mediated JAK-STAT3, PI3K-AKT/mTOR, caspase and NF-κB signaling pathways were among the most downregulated pathways. The full list of pathways from MSigDB 2020 Hallmark and Biocarta 2016 signatures are found in Supplmentary Table S4 and S5, respectively.

**Figure 5.**
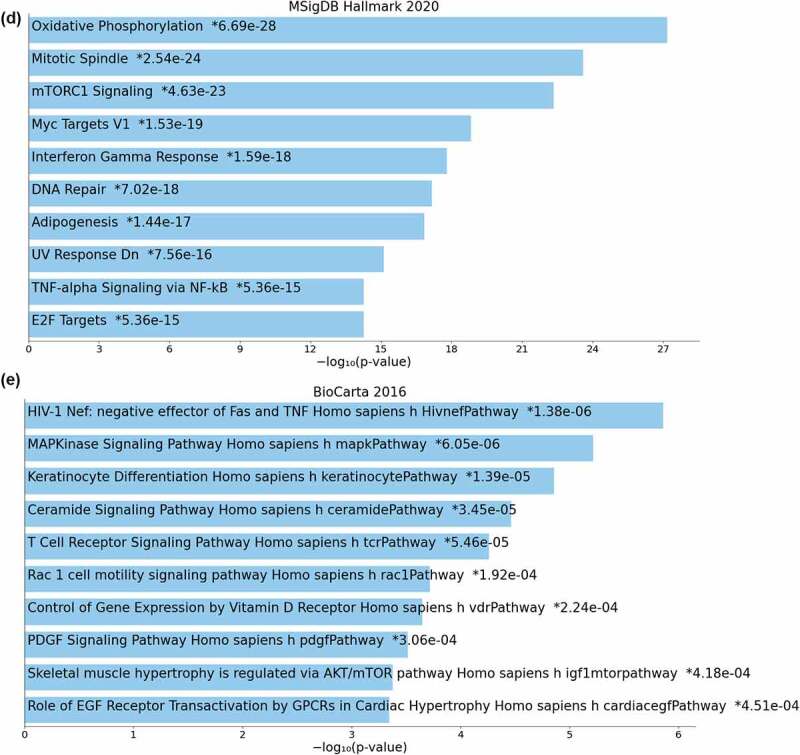
(Continued).

### Pan-cancer gene panel reveals differences in mutational signatures between CD3^high^ and NK^high^ subgroups

To further understand the somatic landscape and spectrum of the two subgroups, we analyzed 34 ccRCC samples (17 tumors: 9 CD3^high^, 8 NK^high^ cases and 17 paired healthy adjacent renal tissue) using a custom-designed targeted sequencing panel covering 986 cancer associated genes and additional intronic cancer hotspots.

As a result, we identified 80 potentially relevant variants from 70 genes recurrently mutated in more than one sample. The *VHL* gene was the most commonly mutated (5 out of 17 cases, 29%), as expected from previous literature^1,5,7^ (Figure 6a). The mutated genes were organized and sorted by their mutational frequencies and annotated according to the CD3^high^ (orange) or NK^high^ groups (yellow) (Figure 6a, middle bar). The majority of the variants in both subgroups harbored missense mutations, particularly small nucleotide polymorphisms (SNPs). Out of 43 mutations in the CD3^high^ subgroup (n = 9): 33 were missense mutations, 4 nonsense mutations, 5 frameshift deletions, and 1 frameshift insertion (Figure 6b). The NK^high^ subgroup (n = 8) harbored a total of 37 mutations: 28 missense mutations, 5 frameshift deletions, 3 nonsense mutations, and 1 frameshift insertion (Figure 6c). In the CD3^high^ subgroup, the *VHL* (33%) and *PBRM1* (33%) genes were the most frequently mutated, whereas in the NK^high^ subgroup, *CSMD1* (25%) together with *VHL* (25%) were the top mutated genes (Figure 6b,c). We further analyzed the differentially expressed mutated genes shared between the two subgroups and found that *PBRM1, AFDN*, and *AR* were uniquely mutated in the CD3^high^ subgroup, whereas *CSMD1, ROS1* and *APC* were distinct to the NK^high^ cases (Figure 6d).

**Figure 6.**
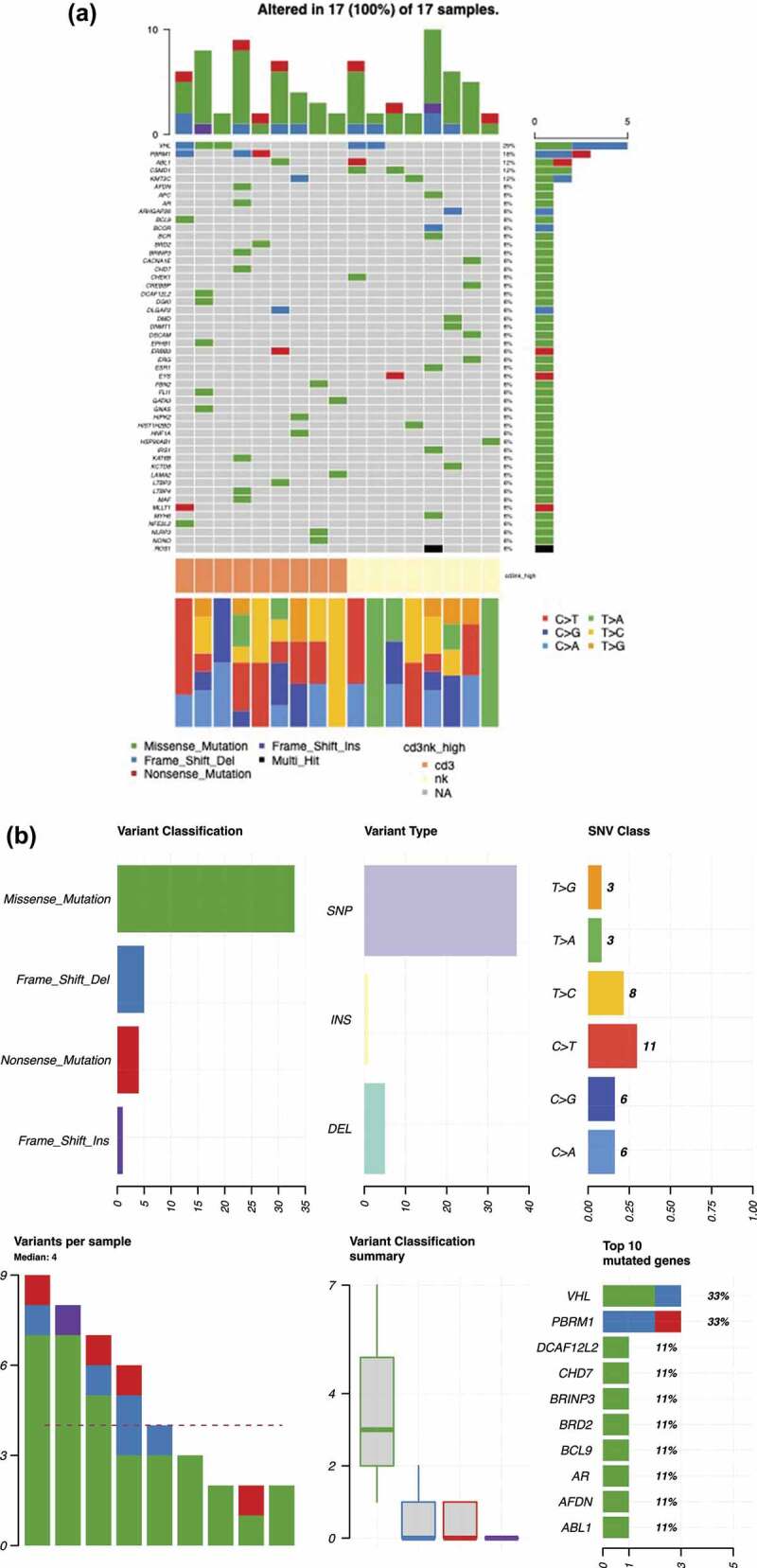
CD3^high^ and NK^high^ subgroups reveal differences in mutational signatures. (a) 17 tumors (9 CD3^high^ and 8 NK^high^) and 17 paired healthy adjacent renal tissues were analyzed using a custom-designed targeted sequencing panel covering 986 cancer associated genes and additional intronic cancer hotspots. 80 variants from 70 genes recurrently mutated in more than one sample were identified. The oncoplot shows the overall summary of the mutational distribution in the top 50 mutated genes. The middle color key represents the CD3^high^ (Orange) and NK^high^ (yellow) subgroups. The bottom stacked barplot shows the distributions of SNVs (six transition and transversion categories) for each sample. Side bar plots (top, right) display the log10 transformed Q-values estimated by MutSigCV. Details of the targeted sequencing panel are found in the Supplemental section. (b) Out of 43 mutations in the CD3^high^ subgroup (n = 9), 33 were missense mutations, 4 nonsense mutations, 5 frameshift deletions, and 1 frameshift insertion. The most frequently mutated genes were *VHL* (33%) and *PBRM1* (33%). (c) The NK^high^ subgroup (n = 8) harbored a total of 37 mutations: 28 missense mutations, 5 frameshift deletions, 3 nonsense mutations, and 1 frameshift insertion, with *CSMD1* (25%) and *VHL* (25%) as the top mutated genes.(d) Differentially expressed mutated genes shared between the CD3^high^ and NK^high^ subgroups. Fisher’s exact test was used to find the genes mutated in a minimum of two samples in at least one of the subgroups for the analysis.

**Figure 6.**
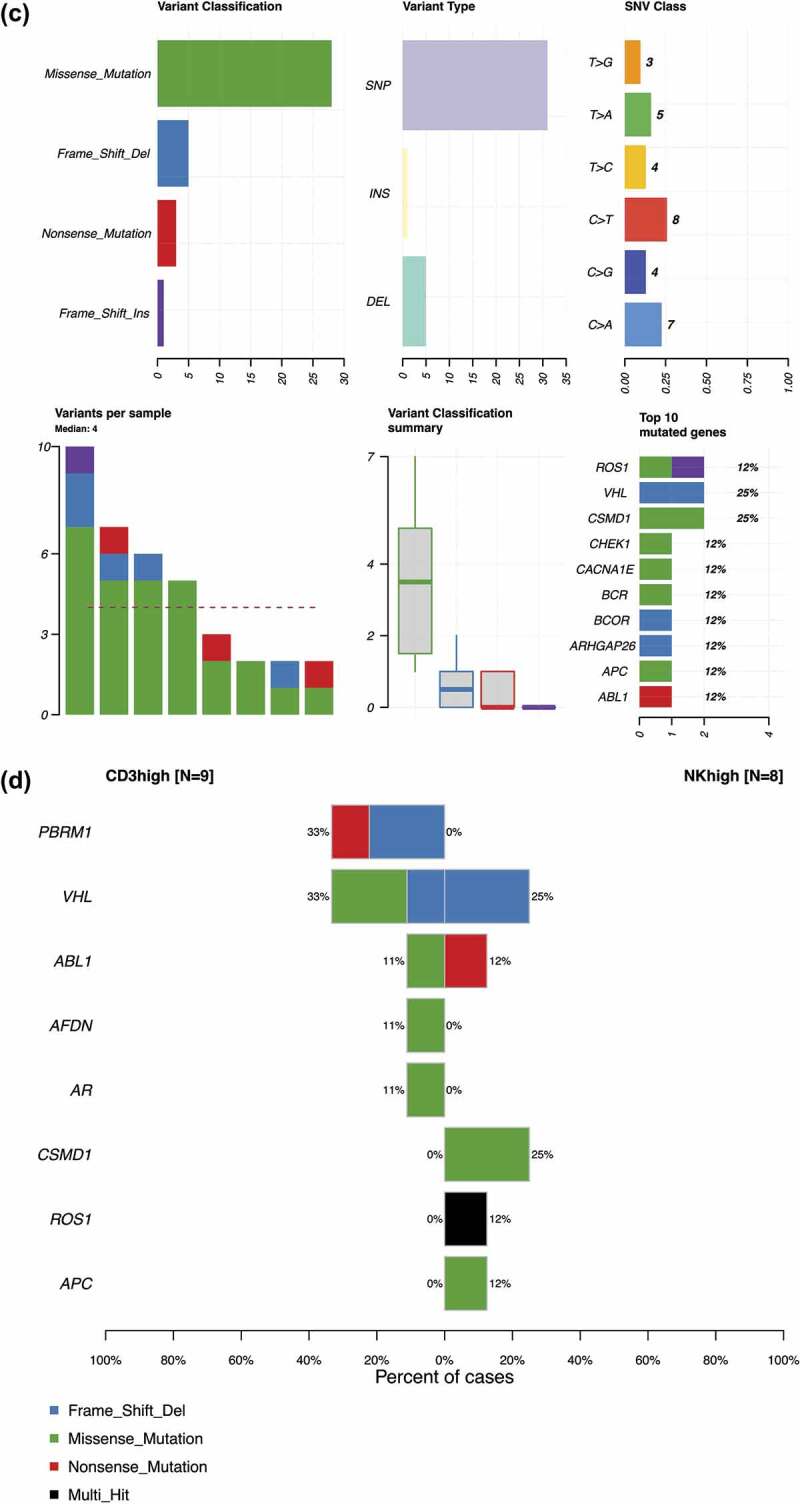
(Continued).

## Discussion

Several clinical, genomic, and molecular studies have shown that RCC is a lymphocyte-rich, immunogenic tumor.^5,13,34,35^ In other solid tumors such as melanoma, head and neck, breast, bladder, and ovarian cancer, CD8+ T cell infiltration is a marker of good prognosis and better overall survival.^35,36^ On the contrary, the infiltration of CD8+ T cells has been associated with poor clinical outcome in RCC.^15,18,35^ Our results suggest that RCC tumors have two distinct immune phenotypes, whereupon T cell rich (CD3^high^) tumors are associated with higher lymphocytic infiltration and expression of PD-1, while NK cell dominant (NK^high^) tumors have a lower abundance of intratumoral lymphocytes.

The immune profile of RCC tumors has also been analyzed in previous studies using flow cytometry, mass cytometry, and immunohistochemistry.^6,12,19,27^ In accordance with these studies, our results show that T cells are the dominant immune cell type in ccRCC; however, in approximately half of the tumors, NK cells are also abundant, consisting ~20% of the lymphocytes.^12,27,28,37^ Although the median lymphocyte count was lower in NK^high^ tumors, some cases had a high lymphocyte count and co-infiltration of CD3+ T cells. Thus, it is the milieu in the lymphocyte compartment that make up the CD3^high^ and NK^high^ subgroups in our study, not the absolute lymphocyte count. Furthermore, all oncocytoma cases clustered to the NK^high^ group, in accordance with earlier findings showing a greater abundance of NK cells in benign tumors (oncocytoma) than in ccRCC.^27,38^ The higher percentage of tumor infiltrating NK cells has also been related to better survival in RCC.^27^ As we acquired a prospective collection of RCC tumors, the clinical follow-up was not long enough to determine possible differences in the survival between patients with CD3^high^ and NK^high^ tumors, and it should be noted that all our cases were treatment-naïve nephrectomy operated patients. Baseline clinical variables such as tumor grade, TNM staging, and WHO classification did not differ between the two subgroups.

Overall, the expression of PD-1 was lower in NK cells compared to T cells, in line with recent findings from *ex vivo* human and mouse models showing minimal PD-1 expression in active NK cells.^39^ NK cell regulation and signaling occur from multiple pathways as opposed to a competitive, antigen-triggered pathway of costimulatory and inhibitory receptors in T cells, and several clinical trials utilizing other (non-PD-1 based) therapies aimed at enhancing the function of NK cells are underway.^24^

The asset in our study are the matched samples including the tumor, adjacent healthy kidney tissue, and peripheral blood (PB). In the PB samples, we did not find a similar dichotomy as in the tumors (CD3^high^/NK^high^ subgroups), and the proportions of the PB T and NK cells did not correlate with the tumor tissue. Overall, our findings suggest that immune phenotypes within the tumor and those in circulation greatly differ; furthermore, tumor infiltrating T and NK cells have a more mature and potentially less functional immune phenotype. Typically, LAG-3 and PD-1 expressions were greater in the tumor, while CX3CR1, CXCR3, and CD27 expressions were lower compared to the PB levels (Supplementary Table S3). Recent studies by Julià and colleagues have explored the changes in blood immune cell subsets after anti-PD-1 therapy and found that an increased frequency of central memory CD4+ T cells and leukocyte count was associated with response, whereas increases in PD-L1+ NK cells and naïve CD4+ T cells were associated with a lack of response,^40^ fueling the need for improved biomarker studies that provide further insight to the circulating lymphocytes in RCC.

Also, when comparing the tumors with the matching healthy renal tissues, we noticed that immune checkpoint receptors (LAG-3 and PD-1) were highly expressed in the tumor compared to the healthy kidney. The total amount of lymphocytes within the tumor was increased only in the CD3^high^ tumors whereas in NK^high^ tumors, the proportion of lymphocytes was similar to those in the matching healthy kidney tissues. Although tumor samples are considered as quite heterogenous tissues, we noticed that immune cell abundancies and phenotypes were strongly matched in the two independent regions of the tumor. However, it should be taken into account that this study includes tumor samples only taken from the tumor core and enzymatically dissociated (thus excluding the peritumoral regions), which in part explains the similarities in immunophenotypes from the independent tissue regions. Further spatial immunoprofiling studies that reflect the intact tumor, and its environment are needed to fully understand the immunological heterogeneities, such as in the recent work by Brück et al,^41^ whereby immunoprofiling was carried out together with image analysis in metastatic RCC.

The somatic mutation landscape of ccRCC has been well characterized, and *VHL, PBRM1, SETD2*, and *BAP1* are the most commonly mutated genes.^9,42^ The mutation profile observed in our patients was well in line with previous findings, and *VHL* and *PBRM1* were among the most commonly mutated genes. When the CD3^high^ and NK^high^ subgroups were independently analyzed, we observed a difference in the mutation profiles. In both cases, *VHL* was mutated in approximately one third of the cases (CD3^high^ 38% and NK^high^ 25%) (Figure 6b,c), but *PBRM1* mutations were only observed in the CD3^high^ group (33% of the cases) (Figure 6b). Recent studies have suggested that in ccRCC, *PBRM1* mutations are associated with better responses to anti-PD1 therapy.^43,44^ The increased lymphocyte infiltration and higher PD-1 expression in the CD3^high^ subgroup of our cohort fits well with these observations. Similarly, as shown before with *PBRM1* mutated tumors, the transcriptomic profile of CD3^high^ group was characterized with increased immune signaling pathways (IFN-γ, TNF-α via NF-κB, and T cell receptor signaling) and kidney metabolism (fatty acid metabolism, oxidative phosphorylation). Although a low expression of *CD274 (PD-L1)* was observed in the CD3^high^ subgroup, the monophasic marker is deemed unfavorable for clinical use in RCC and urothelial cancers.^45^ Further understanding of the role of PD-L1 and the mechanisms involved in the inhibition of T cell immune responses are needed as the treatment failures are still poorly understood.^12^ In addition, the solute carrier family of genes (*SLC5A12, SLC22A7*) were among the most differentially expressed genes between the CD3^high^ and NK^high^ subgroups.^12,45^ Recent studies have proposed that the dysregulation of solute carrier family of genes play diagnostic and prognostic roles in ccRCC, where among increased *SLC22A7* expression was associated with predicting improved overall and disease-free survival in ccRCC patients.^46,47^ Further studies focusing on the interplay of the immune, metabolic and tumor-specific signaling pathways will add to our understanding of the transcriptional changes in ccRCC.

In summary, our study demonstrates substantial differences in the immune phenotype of RCC tumors from paired healthy kidney tissues and peripheral blood samples, as well as underlines the valuable use of tumor samples in biomarker studies. Furthermore, immunoprofiling revealed two distinct subtypes of ccRCC tumors: CD3^high^ and NK^high^ groups. The CD3^high^ subgroup was associated with increased PD-1 expression and a unique mutation profile, which possibly define those patients that may benefit from current anti-PD1 therapies. Further studies are needed to clarify whether the NK^high^ subgroup will benefit from alternative treatment options targeting the NK cells.

## Supplementary Material

Supplemental Material
