# Supplementary material for: T and NK cell abundance defines two distinct subgroups of renal cell carcinoma
Source: Oncoimmunology. 2022 Jan 4;11(1):1993042. doi: 10.1080/2162402X.2021.1993042 (PMC8741293; doi:10.1080/2162402X.2021.1993042)
Supplement: Supplemental Material [file KONI_A_1993042_SM1805.zip › rcc_mhl_supp_fin.pdf]

Supplemental Methods

Bulk RNA sequencing

Total RNA was isolated and checked for their quality. RNA extractions, quality control, whole transcriptome library preparations, complementary DNA library, and sequencing was performed according to the Illumina Novaseq 6000 standard protocol by Novogene Bioinformatics Technology Cooperation (https://en.novogene.com/). Ribosomal RNA (rRNA) was removed, and strand-specific sequencing libraries were generated following manufacture's recommendations. Long non-coding RNA (lnc-RNA) was extracted for each sample and further sequenced in order to detect only those transcripts that were over 200 nucleotides in length and did not encode a protein involved in multiple biological processes. The library preparation however allowed the enrichment and gene expression profiling of both coding and non-coding transcripts.

Bulk RNA sequencing data preprocessing

Analysis of RNA-sequencing data was performed mainly as previously described[1]. Briefly, paired-end reads were aligned to human reference genome build 38 (EnsEMBL v82) using STAR2[2] with the guidance of EnsEMBL v82 gene models. Analysis was done using default 2-pass per-sample mapping settings, except for the overhang of the splice junctions that was set to 99. Reads were sorted by coordinate using the SortSAM, PCR duplicates were marked with the MarkDuplicate module of the Picard toolkit[3], feature counts were generated using SubRead[4] , feature counts were converted to expression estimates using Trimmed Mean of M-values (TMM) normalization[5] , and lowly expressed genomic features with a CPM value ≤1.00 in less than half of samples removed. Differential expression testing was performed using the edgeR software[6] and DESeq2[7] (1.29.16) R package.
In the statistical testing, comparisons between subject groups included factors for sequencing kit. The p-values were adjusted with Storey's Q-value for multiple comparisons[8]. Differentially expressed genomic features were determined with Q ≤0.05 cutoff value. In data visualization and pathway analyses, we used batch-corrected CPM data for library preparation kit effect. Batches were corrected using the removeBatchEffect function in the package limma[9]. Clustering of gene expression profiles

was performed with both genes and samples using the Euclidean distance and Ward linkage method. Differentially expressed genomic features were determined using $p<0.1$ as a cutoff value.

Gene set enrichment pathway analysis

Pathway enrichment analysis was done using the Broad Institute's MSigDB (2020) hallmark[10] and Biocarta (2016)[11] gene sets. In the GSEA analysis, a pre-ranked gene list was prepared by sorting genes by their log-fold change in the batch corrected CPM data. GSEA analysis was then performed using default values. Fold change >2 was used as a threshold to interpret analysis output.

Pan-cancer gene panel

50ng of genomic DNA (gDNA) was processed from 20 tumors and their matching adjacent healthy tissue samples according to Twist Custom Panel EF Multiplex Complete kit (Twist Bioscience,San Francisco, CA, USA) with the following modifications: 4µl of xGen Dual Index UMI Adapter (Integrated DNA Technologies, Coralville, IA, USA) was used per sample. Libraries were quantified for custom panel capture using LabChip GX Touch HT High Sensitivity assay (PerkinElmer, Waltham, MA, USA). Eight samples were pooled for each target enrichment reaction. Twist custom panel (6.2MB) probes were used in the capture. The custom panel targets 986 cancer associated genes and additional intronic cancer hot spots. The captured library pools were quantified for sequencing using KAPA Library Quantification Kit (KAPA Biosystems, Wilmington, MA, USA) and LabChip GX Touch HT High Sensitivity assay.

Libraries were sequenced on NovaSeq6000 system (Illumina, San Diego, CA, USA) using SP flow cell and standard workflow (Illumina, San Diego, CA, USA). Read length for the paired-end run was 2x101. Reads were trimmed to PE101 for the analysis.

Variant analysis

The sequencing data was pre-processed for low quality, adapter sequences, and short read length using the Trimmomatic[12] software. Paired-end reads passing filters were then aligned to human reference genome build 38 (EnsEMBL v82) using BWA-MEM[12], alignments were sorted by coordinate using

SortSAM, and PCR duplicates were marked with the MarkDuplicate module of the Picard toolkit[3]. Default parameters were used. Calling of variants employed Genome Analysis Toolkit (GATK) toolset13[12] and was based on the GATK somatic short variant best practice (version 3.5), supplemented with the estimation of the cross-sample contamination level and filtering of the 8-oxoguanine and deamination artefacts with GATK4 CalculateContamination, CollectSequencingArtifactMetrics, and FilterByOrientationBias tools. Variant calling was done using the Genome Analysis Toolkit (GATK) toolset. The GATK resource files were converted from GRCh37 to GRCh38 using CrossMap[13] and the EnsEMBL chain files were downloaded from the EnsEMBL database. Variant calls were finally normalized using bcftools[14]. Variants were annotated and filtered using the Annovar tool[15] against the RefGene database. First, MuTect2 filters with a TLOD ≥ 6.3 or a TLOD ≥ 5.0 and supported by five or more independent COSMIC[16] were applied to all samples and variants other than those passing were filtered. For the trinucleotide profile, variant data were then filtered for false-positives by removing variants in intronic regions and with coverage ≤ 10, variant quality value ≤ 40, variant allele frequency ≤ 2.00%, strand odd ratio for SNVs ≥ 3.00, and strand odd ratio for indels ≤ 11.00, minor allele frequency ≥ 1% in the EPS and 1KG databases, minor allele frequency ≥ 1% in general, Finnish, and Non-European ExAC databases, and minor allele frequency ≥ 0.01% in general, Finnish, and Non-European ExAC databases and supported by less than six independent COSMIC samples. For functional analyses, the previous variant call set was filtered further by removing synonymous mutations and non-frameshift variants. deconstructSigs[17] software was used to identify mutational signatures, using the with default parameters and cancer profiles downloaded from the COSMIC website in September 2017. Structural rearrangements were called with Manta[18] from the same alignment files subjected to somatic short variant calling.

The EnsEMBL to UCSC chromosome nomenclature conversions were done using the mapSeqlevels from the GenomeInfoDb package[19]. Driver gene candidates were identified with the use of MutSigCV60 and Oncodrive-fm61. MutSigCV was executed using the default mutation rate covariate after fixing its gene names to current nomenclature with maftools62, a hg38-compatible coverage file generated with the CovGen tool, and the default mutation type dictionary file after addition of Annovar variant categories into it. For Oncodrive-fm, functional impact scores of missense mutations

were gathered from the PolyPhen2 (HumVar), SIFT, and MutationAssessor annotations of Annovar outputs. Scores were manipulated in accordance with recommendation61 and Oncodrive-fm executed with default setting except for setting the minimum number of mutations per gene to 3. In order to call the variants, the tumor-normal variant calling strategy was used. The variants were filtered against a panel of normals consisting of variants detected in two or more DNA samples of 10 healthy adjacent renal tissues from the matching tumor samples.

Multiplexed immunohistochemistry (mIHC) of whole tissue slides and image processing

FFPE samples from 11 tumors and 11 of the matching adjacent healthy kidney tissue samples were deparaffinized in xylene and rehydrated in a series of graded ethanol solution. Heat-induced epitope retrieval (HIER) was performed in 10mM Tris-HCl-1mM EDTA buffer (pH 9) in +99°C for 20min. Peroxide activity was blocked using 0.9% H_2O_2 for 15min, with subsequent application with 10% normal goat serum (TBS-NGS) for 15min. The following antibodies (dilution) and staining reagents were used: (panel 1) FOXP3 (1:200) TSA488 (Abcam #ab20034), CD3 (1:750) TSA555 (Thermofisher #MA5-14482), CD4 (1:50) AF647 (Abcam #ab133616), CD8 (1:300) AF750 (Dako #M7103; (panel 2) CD45 (1:200) AF647 (Dako #M0701), CAIX (1:200) AF750 (Novus Biologicals #NB100-417), E-cadherin (1:200) AF750 (Cell Signaling Technnology #3195), pan-cytokeratin (1:200) AF750 (Abcam #9377). The staining procedures highlighted from previous literature[20] were applied for this experiment. All mIHC images were acquired with the Axio Scan.Z1 Digital Slide Scanner (Zeiss) using the 20X (0.8NA) plan apochromat objective (Zeiss), ORCA-Flash 4.0 V2 Digital CMOS camera (Hamamatsu Photonics K.K.) and Colibri.7 LED light source (Zeiss) and were converted to JPG format. The staining was performed in two rounds. The acquired images were exported in jpeg-compressed tiff format and registered using 2-dimensional phase correlation method[21].

Supplemental figure legends

Supplementary Figure S1 ccRCC heatmap and comparison of immune cells between oncocytoma and other malignant cases

A. Heatmap of ccRCC cases according to the intratumoral CD3+ T and NK cell abundance using Spearman correlation and ward.D2 clustering methods. Bottom panels I and J show tumor samples that have been used for bulk RNA sequencing and the pan-cancer gene panel, respectively.
B. Intratumoral immune cell subsets of benign oncocytoma cases (n=5) vs malignant (clear cell, chromophobe, papillary) RCC subtypes. Oncocytomas carried a higher proportion of NK cells as compared to other malignant tumors. Colors indicate different subtypes of RCC (red = clear cell, orange = chromophobe, turquoise = papillary, yellow = oncocytoma). Mann-Whitney U test: ns, not significant, *, $p<0.05$; **, $p<0.01$.

Supplementary Figure S2 Multiplexed immunohistochemistry (mIHC) of tumor whole tissue slides

A. and B. Two representative ccRCC whole tissue slide samples stained for DAPI, CD3, CD4 and CD8, showing (A.) a high infiltration of T cells (CD3^{high}) and (B.) low infiltration (NK^{high}), respectively. Pathologist-confirmed HE stainings of the corresponding tumor samples (far right) supports the degree of infiltration observed in the mIHC analysis.
C. Scatter dot plots showing the proportion of immune cells (CD3+, CD3+CD4+, CD3+CD8+ and CD3+CD4+FOXP3+) from the surface area of the DAPI+ cells between the CD3^{high} and NK^{high} subgroups. Mann-Whitney U test: ns, not significant, horizontal lines show the median and range.

Supplementary Figure S3 Flow gating strategies for two representative cases (lymphocyte-poor and -rich)

All cells beyond the axis of origin were labeled as "live cells" and represent the total population of obtained cells. From the CD45+ population, the lymphocytes were gated, and the different lymphocytic subsets gated from the lymphocyte population.

A. Healthy adjacent kidney tissue (lymphocyte-rich)
B. Healthy adjacent kidney tissue (lymphocyte-poor)
C. Tumor tissue (lymphocyte-rich)
D. Tumor tissue (lymphocyte poor)
E. Gating strategy of one representative tumor case regarding the different immune subsets, as well as an example (PD-1) of the marker expressions for each subset.

Supplementary Figure S4 Statistical analysis of clinical parameters

A. Comparison of gender and WHO ISUP 2016 tumor grade between the $CD3^{high}$ and NK^{high} subgroups. Patients in the NK^{high} group were slightly older than those in the $CD3^{high}$ group. Mann-Whitney U test: *, $p<0.05$.
B. Comparison of the presence of necrosis between the $CD3^{high}$ and NK^{high} subgroups. Mann-Whitney U test: ns, not significant.

Supplementary Figure S5 Changes in the lymphocyte subsets, LAG-3 and PD-1 expressions between the $CD3^{high}$ and NK^{high} tumor-healthy pairs

A. Pearson correlation of lymphocyte subsets between tumor (T) and matching healthy kidney tissue (H) according to the $CD3^{high}$ and NK^{high} subgroups. In the $CD3^{high}$ subgroup, the tumor and healthy tissue (i) lymphocytes ($R=0.92$, $p=5x10^{-5}$) as well as the (vii) CD8+ T cells ($R=0.67$, $p=0.05$) correlate well with each other.

B. LAG-3 and PD-1 marker expressions between the tumor and healthy in the $CD3^{high}$ and NK^{high} subgroups: CD4+ LAG-3 ($CD3^{high}$ subgroup $p=0.01$, median 2.8% vs 0.2%) and CD8+ LAG-3 ($CD3^{high}$ subgroup $p=0.02$ median 6.4% vs 0.9%). No differences were observed in NK

LAG-3 expression between the two subgroups. Kruskal-Wallis statistical test: ns, not significant, *, $p<0.05$.

Supplementary Figure S6 Differences in the marker expression between the tumor and adjacent healthy tissue

The proportion of CD57 (A), CD27 (B), CXCR4 (C) and NKG2D (D) positive CD4+ and CD8+ T cells and NK cells between tumor (T) and healthy (H) tissue. All statistical analyses were done using Mann-Whitney U test: ns, not significant, *, $p<0.05$; **, $p<0.01$; ***, $p<0.001$.

Supplementary Figure S7 Phenotype of two alternate regions of the tumor

A-F. To take account the spatial heterogeneity of the tumors, two alternate regions (t1, t2) of the tumor sample were independently phenotyped. Strong correlations between the immune cell abundancies of the distinct regions were observed: lymphocytes R=0.88 (p=6.2x10-5), CD3+ R=0.84 (p=0.00018), CD4+ R=0.92 (p=2.8x10-6), CD8+ R=0.57 (p=0.032), NK R=0.55 (p=0.043), and NKT cells R=0.55 (p=0.043). T-test was used to statistically compare the regions.

G. Sample processing strategy: t1 and t2: Independent phenotyping of two spatially different regions of the same tumor; t3: dissociation, sample processing and frozen; t4: processed for FFPE blocks and HE staining. Image created with BioRender.com.

Supplementary Figure S8 Comparison of lymphocyte subsets between the tumor and matching PB samples

A. Pearson correlation of tumor (T) and peripheral blood (PB) lymphocyte subsets. No differences were observed between the intratumoral and circulating lymphocytes.
B. The PB samples encompass a greater proportion of lymphocytes than the tumor tissues ($p<0.0001$, median 15.8% vs 1.9%). The proportion of CD3+, CD4+, CD8+ T and NK cells from the total lymphocyte population in the tumor and peripheral blood (PB) samples. Mann-Whitney U test: ns, not significant, **** $p<0.0001$.

C. The proportion of LAG3 positive CD4+, CD8+ T and NK cells in tumor (T) and peripheral blood (PB) samples in the CD3^{high} and NK^{high} subgroups. Kruskal-Wallis test: *, p<0.05; **, p<0.01; ***, p<0.001; ****, p<0.0001.

D. The proportion of PD1 positive CD4+, CD8+ T and NK cells in tumor (T) and peripheral blood (PB) samples in the CD3^{high} and NK^{high} subgroups. Kruskal-Wallis: *, p<0.05; **, p<0.01; ***, p<0.001; ****, p<0.0001.

Supplementary Table S1 List of flow cytometry antibodies

Supplementary Table S2 Full list of clinical parameters and patient medical history

Supplementary Table S3 Table comparing tumor, matching PB and healthy tissue according to the lymphocyte subsets and marker expressions

All median and range values for the immune subset population (CD3, CD4, CD8, NK, NKT) other than the lymphocytes (from total "live cell" population) have been calculated from the lymphocyte population. Kruskal-Wallis test was used for the comparisons between T vs PB, T vs H and PB vs H, as well as each expression marker. T = tumor, PB = peripheral blood, H = healthy. Dunn's correction for multiple comparisons" with an alpha threshold and confidence level of 0.05 was used to compare three or more groups of continuous variables: *, p<0.033; **, p<0.0021; ***, p<0.0002; ****, p<0.0001.

Supplementary Table S4 Table showing GSEA of MSigDB 2020 hallmark pathways

Supplementary Table S5 Table showing GSEA of Biocarta 2016 pathways

References

1. Kumar A, Kankainen M, Parsons A, Kallioniemi O, Mattila P, Heckman CA. The impact of RNA sequence library construction protocols on transcriptomic profiling of leukemia. *BMC Genomics*. 2017;18(1):629-1. doi: 10.1186/s12864-017-4039-1 [doi].

2. Dobin A, Davis CA, Schlesinger F, et al. STAR: Ultrafast universal RNA-seq aligner. *Bioinformatics*. 2013;29(1):15-21. doi: 10.1093/bioinformatics/bts635 [doi].

3. Broad Institute, GitHub Repository. Picard toolkit. http://broadinstitute.github.io/picard/. Updated 2019.

4. Liao Y, Smyth GK, Shi W. The subread aligner: Fast, accurate and scalable read mapping by seed-and-vote. *Nucleic Acids Res*. 2013;41(10):e108. doi: 10.1093/nar/gkt214 [doi].

5. Robinson MD, Oshlack A. A scaling normalization method for differential expression analysis of RNA-seq data. *Genome Biol*. 2010;11(3):R25-r25. Epub 2010 Mar 2. doi: 10.1186/gb-2010-11-3-r25 [doi].

6. Robinson MD, McCarthy DJ, Smyth GK. edgeR: A bioconductor package for differential expression analysis of digital gene expression data. *Bioinformatics*. 2010;26(1):139-140. doi: 10.1093/bioinformatics/btp616 [doi].

7. Love MI, Huber W, Anders S. Moderated estimation of fold change and dispersion for RNA-seq data with DESeq2. *Genome Biol*. 2014;15(12):550-8. doi: s13059-014-0550-8 [pii].

8. Storey JD. A direct approach to false discovery rates. *Journal of the Royal Statistical Society.Series B (Statistical Methodology)*. 2002;64(3):479-498. http://www.jstor.org/stable/3088784.

9. Ritchie ME, Phipson B, Wu D, et al. Limma powers differential expression analyses for RNA-sequencing and microarray studies. *Nucleic Acids Res*. 2015;43(7):e47. doi: 10.1093/nar/gkv007 [doi].

10. Liberzon A, Birger C, Thorvaldsdottir H, Ghandi M, Mesirov JP, Tamayo P. The molecular signatures database (MSigDB) hallmark gene set collection. *Cell Syst*. 2015;1(6):417-425. doi: 10.1016/j.cels.2015.12.004 [doi].

11. Rouillard AD, Gundersen GW, Fernandez NF, et al. The harmonizome: A collection of processed datasets gathered to serve and mine knowledge about genes and proteins. *Database (Oxford)*. 2016;2016:10.1093/database/baw100. Print 2016. doi: 10.1093/database/baw100 [doi].

12. Li H. Aligning sequence reads, clone sequences and assembly contigs with BWA-MEM. *arXiv e-prints*. 2013:arXiv:1303.3997. https://ui.adsabs.harvard.edu/abs/2013arXiv1303.3997L.

13. Zhao H, Sun Z, Wang J, Huang H, Kocher JP, Wang L. CrossMap: A versatile tool for coordinate conversion between genome assemblies. *Bioinformatics*. 2014;30(7):1006-1007. doi: 10.1093/bioinformatics/btt730 [doi].

14. Li H. BFC: Correcting illumina sequencing errors. *Bioinformatics*. 2015;31(17):2885-2887. doi: 10.1093/bioinformatics/btv290 [doi].

15. Wang K, Li M, Hakonarson H. ANNOVAR: Functional annotation of genetic variants from high-throughput sequencing data. *Nucleic Acids Res*. 2010;38(16):e164. doi: 10.1093/nar/gkq603 [doi].

16. Forbes SA, Beare D, Boutselakis H, et al. COSMIC: Somatic cancer genetics at high-resolution. *Nucleic Acids Res*. 2017;45(D1):D777-D783. doi: 10.1093/nar/gkw1121 [doi].

17. Rosenthal R, McGranahan N, Herrero J, Taylor BS, Swanton C. DeconstructSigs: Delineating mutational processes in single tumors distinguishes DNA repair deficiencies and patterns of carcinoma evolution. *Genome Biol*. 2016;17:31-4. doi: 10.1186/s13059-016-0893-4 [doi].

18. Chen X, Schulz-Trieglaff O, Shaw R, et al. Manta: Rapid detection of structural variants and indels for germline and cancer sequencing applications. *Bioinformatics*. 2016;32(8):1220-1222. doi: 10.1093/bioinformatics/btv710 [doi].

19. Arora Sonali, Morgan Martin, Carlson Marc, Pages H. GenomeInfoDb: Utilities for manipulating chromosome names, including modifying them to follow a particular naming style. R package version 1.26.0. https://bioconductor.org/packages/GenomeInfoDb. Updated 2020.

20. Bruck O, Lee MH, Turkki R, et al. Spatial immunoprofiling of the intratumoral and peritumoral tissue of renal cell carcinoma patients. *Mod Pathol*. 2021. doi: 10.1038/s41379-021-00864-0 [doi].

21. B. S. Reddy, B. N. Chatterji. An FFT-based technique for translation, rotation, and scale-invariant image registration. *IEEE Transactions on Image Processing*. 1996;5(8):1266-1271. doi: 10.1109/83.506761.
