# Supplementary material for: T and NK cell abundance defines two distinct subgroups of renal cell carcinoma
Source: Oncoimmunology. 2022 Jan 4;11(1):1993042. doi: 10.1080/2162402X.2021.1993042 (PMC8741293; doi:10.1080/2162402X.2021.1993042)

Supplementary Fig. S1

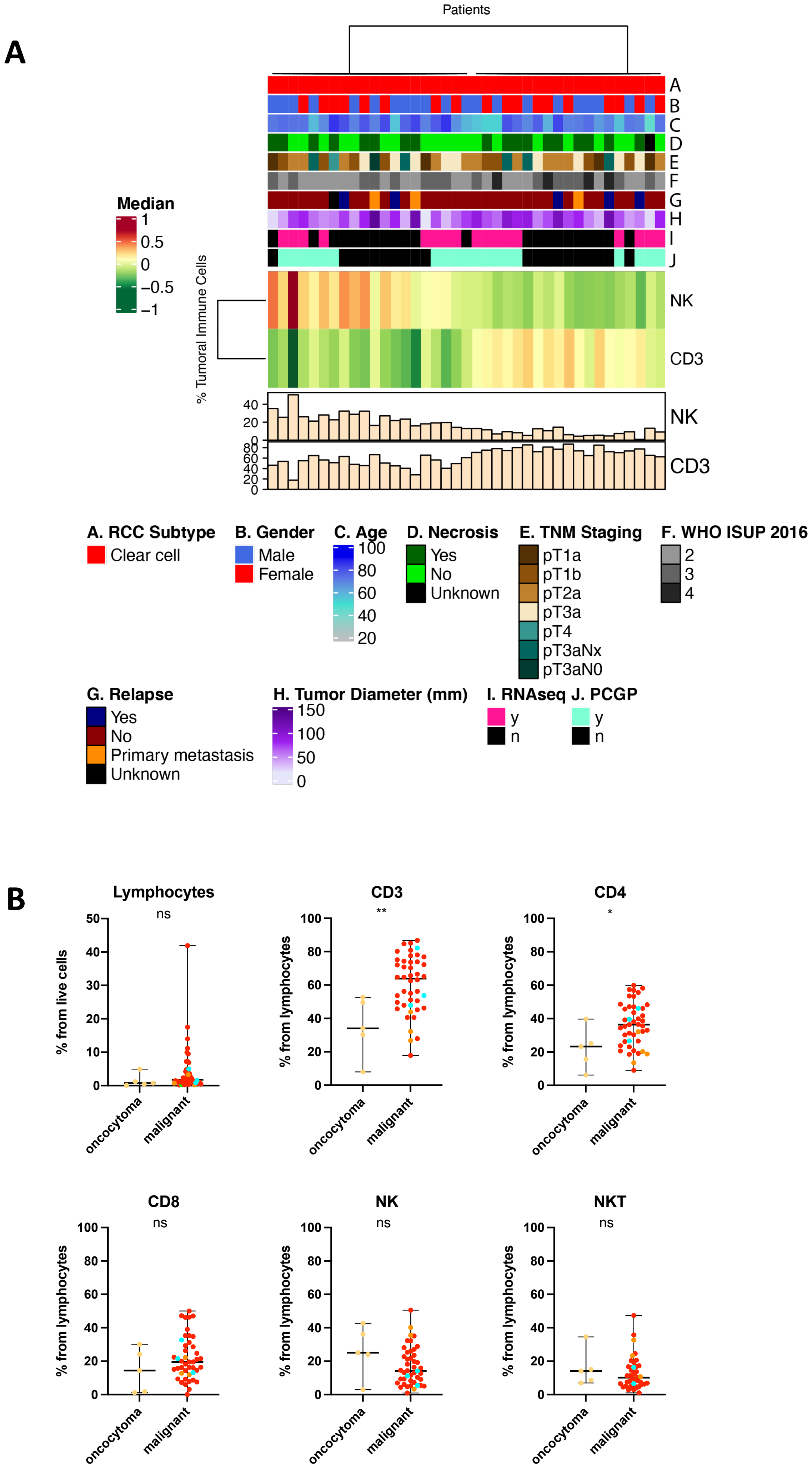

Supplementary Fig. S2

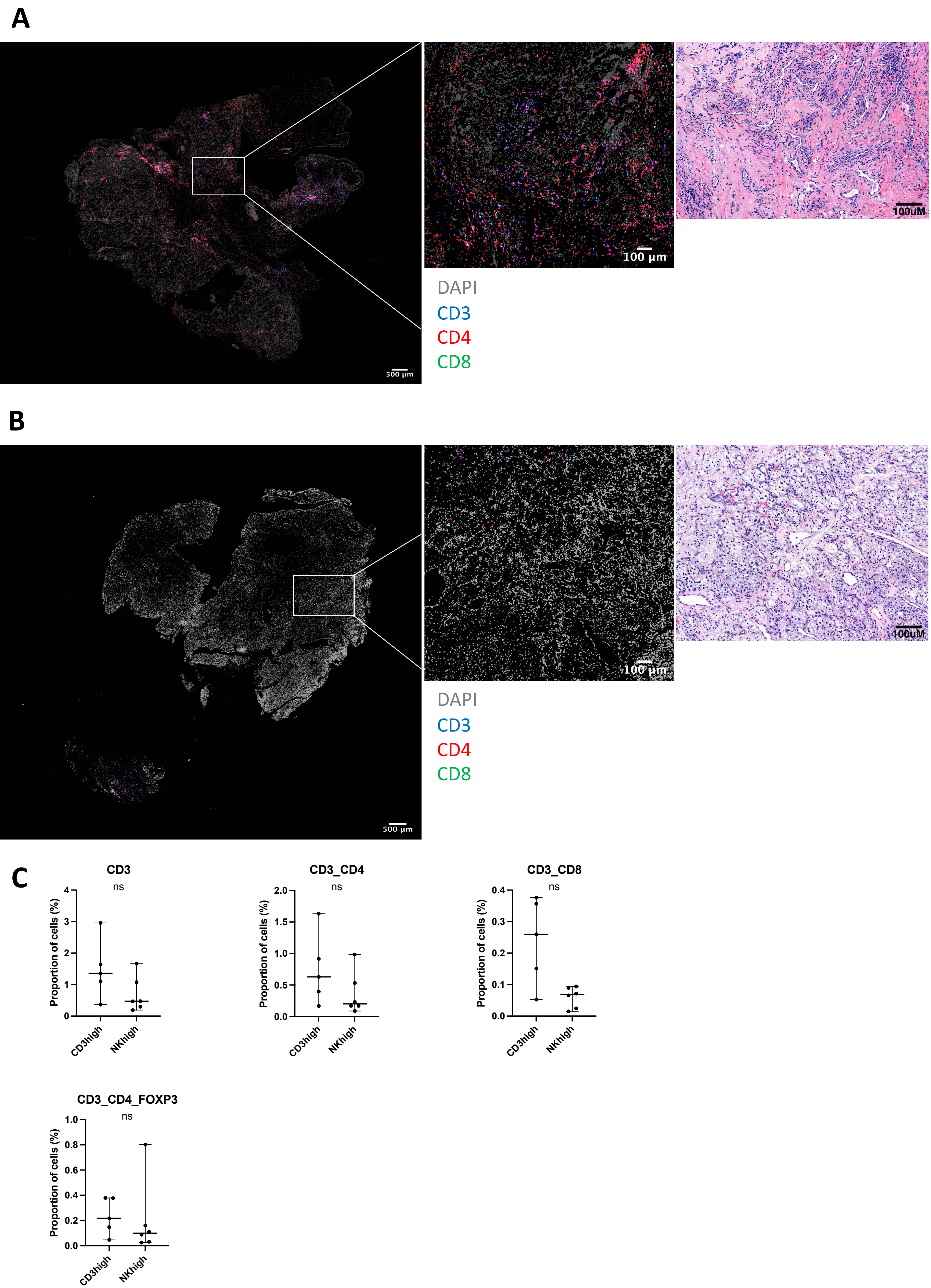

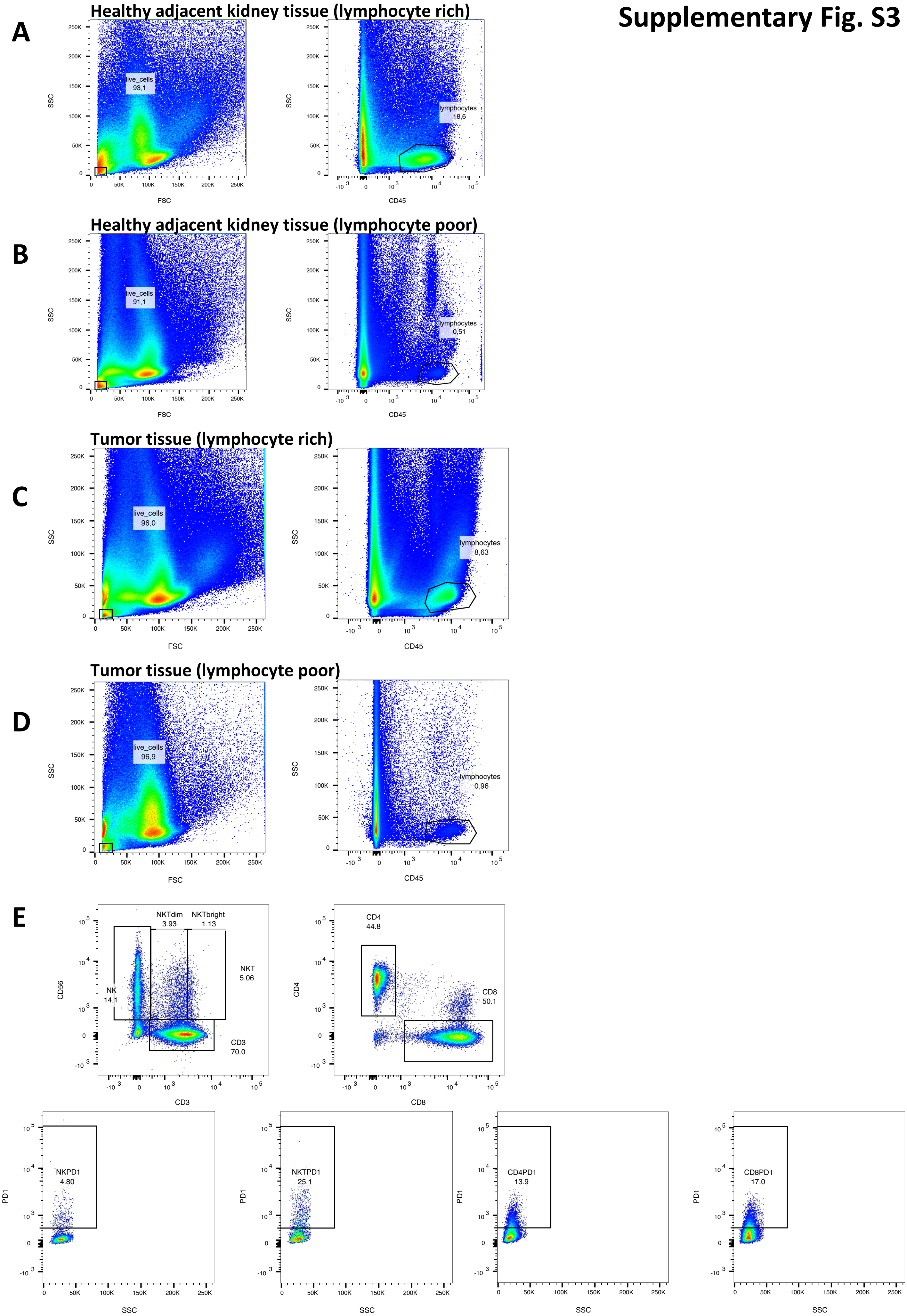
Supplementary Fig. S3
A
Healthy adjacent kidney tissue (lymphocyte rich)
live_cells
93,1
lymphocytes
18,6
SSC
FSC
CD45
B
Healthy adjacent kidney tissue (lymphocyte poor)
live_cells
91,1
lymphocytes
0,51
C
Tumor tissue (lymphocyte rich)
live_cells
96,0
lymphocytes
8,63
D
Tumor tissue (lymphocyte poor)
live_cells
96,9
lymphocytes
0,96
E
NKTdim
3.93
NKTbright
1.13
NKT
5.06
NK
14.1
CD3
70.0
CD56
CD4
44.8
CD8
50.1
NKPD1
4.80
NKTPD1
25.1
CD4PD1
13.9
CD8PD1
17.0
PD1

Supplementary Fig. S4

A

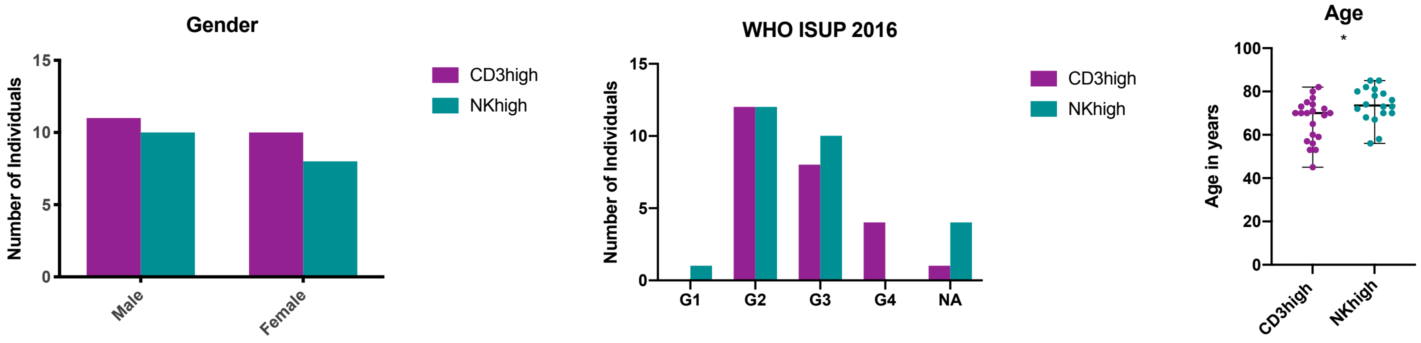

B

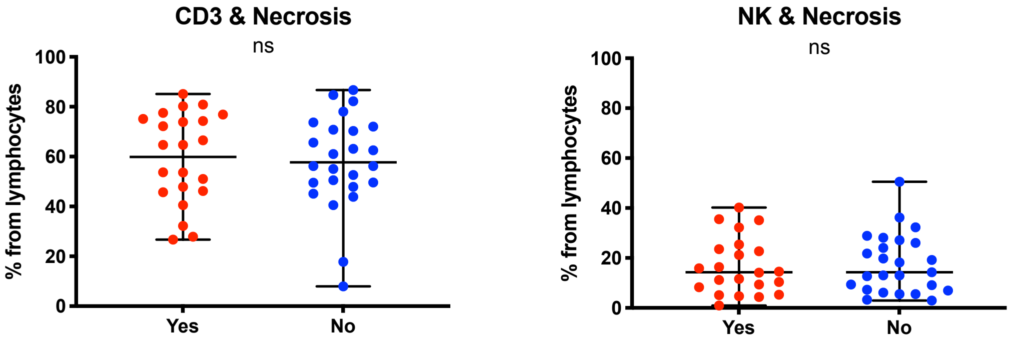

Supplementary Fig. S5

A

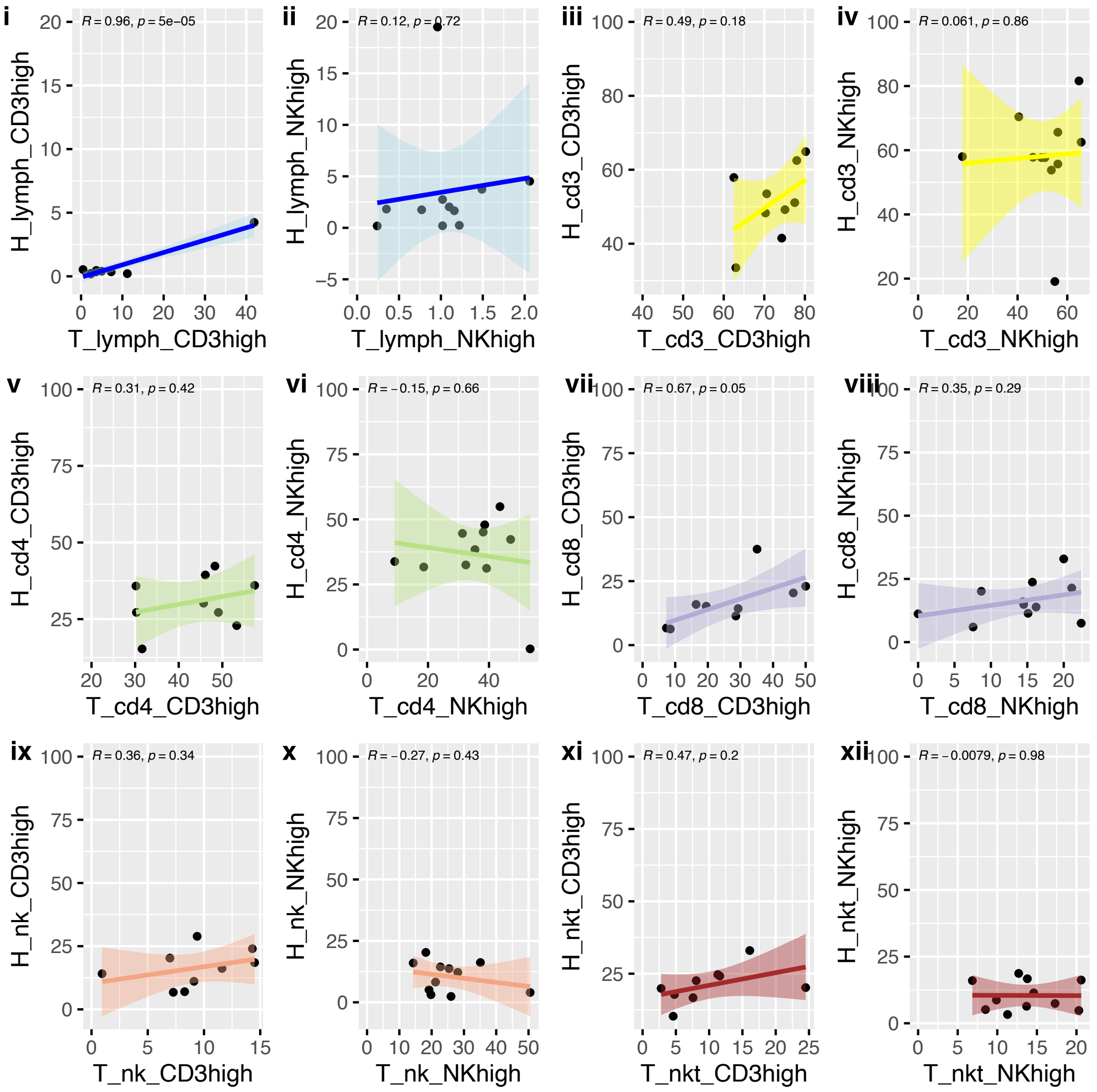

B

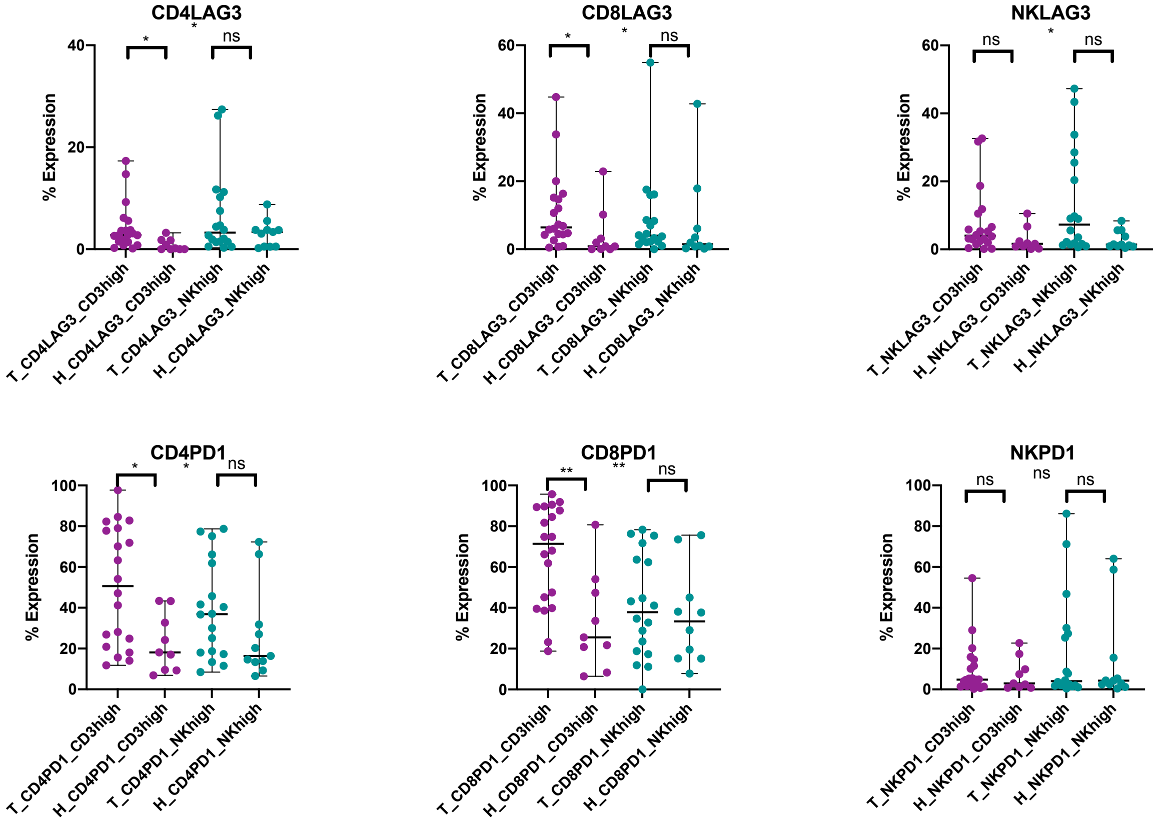

Supplementary Fig. S6

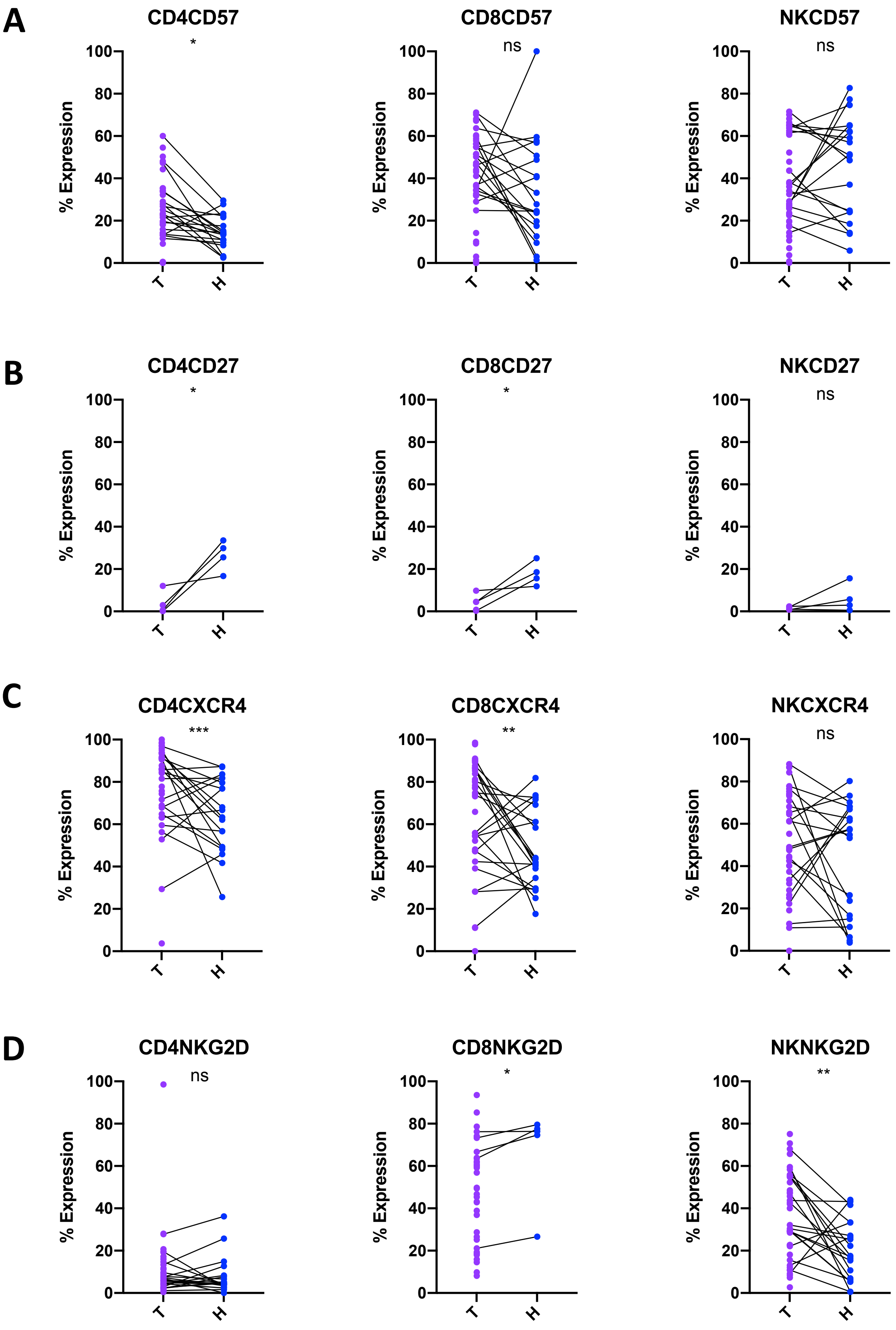

Supplementary Fig. S7

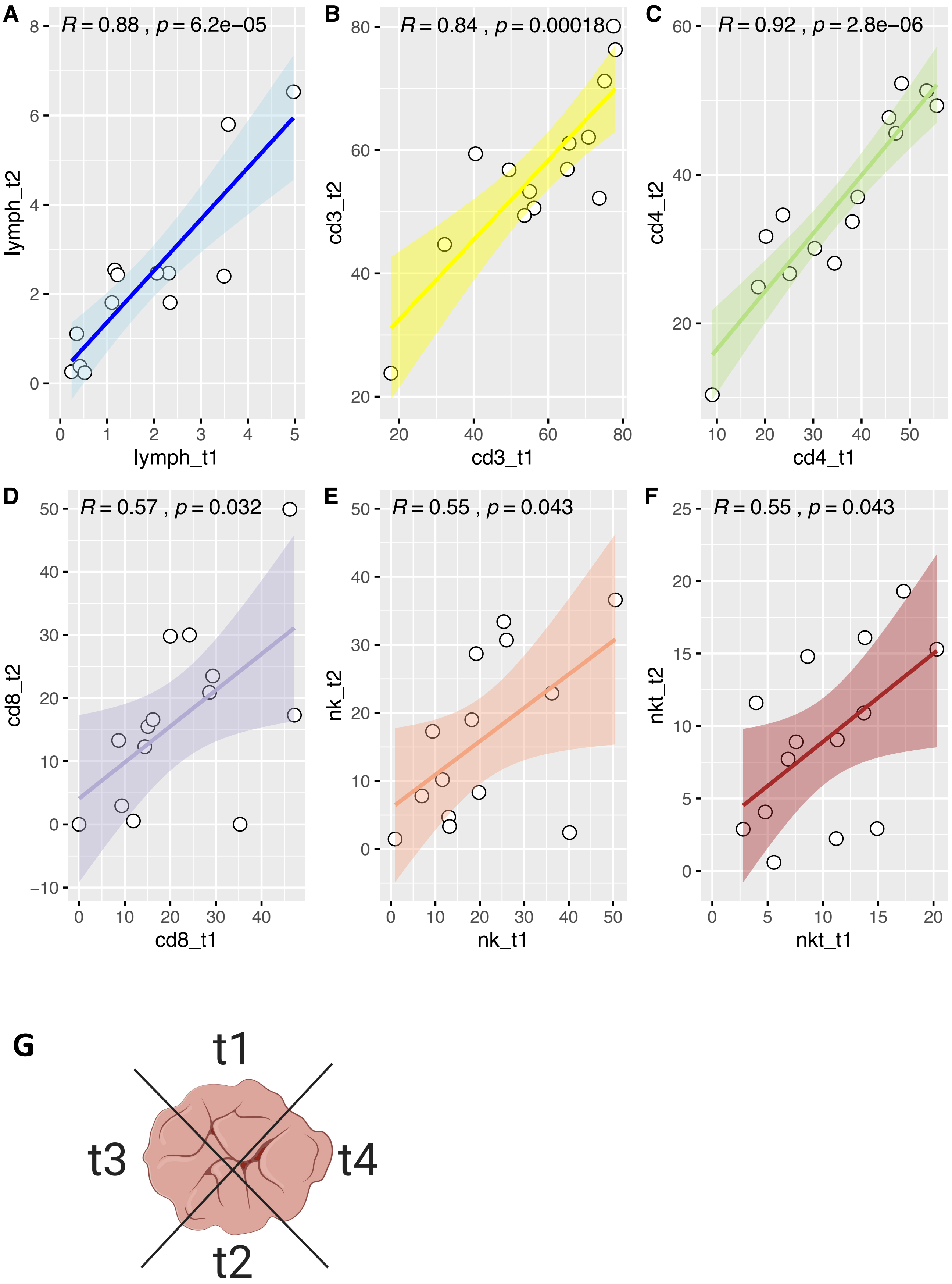

Supplementary Fig. S8

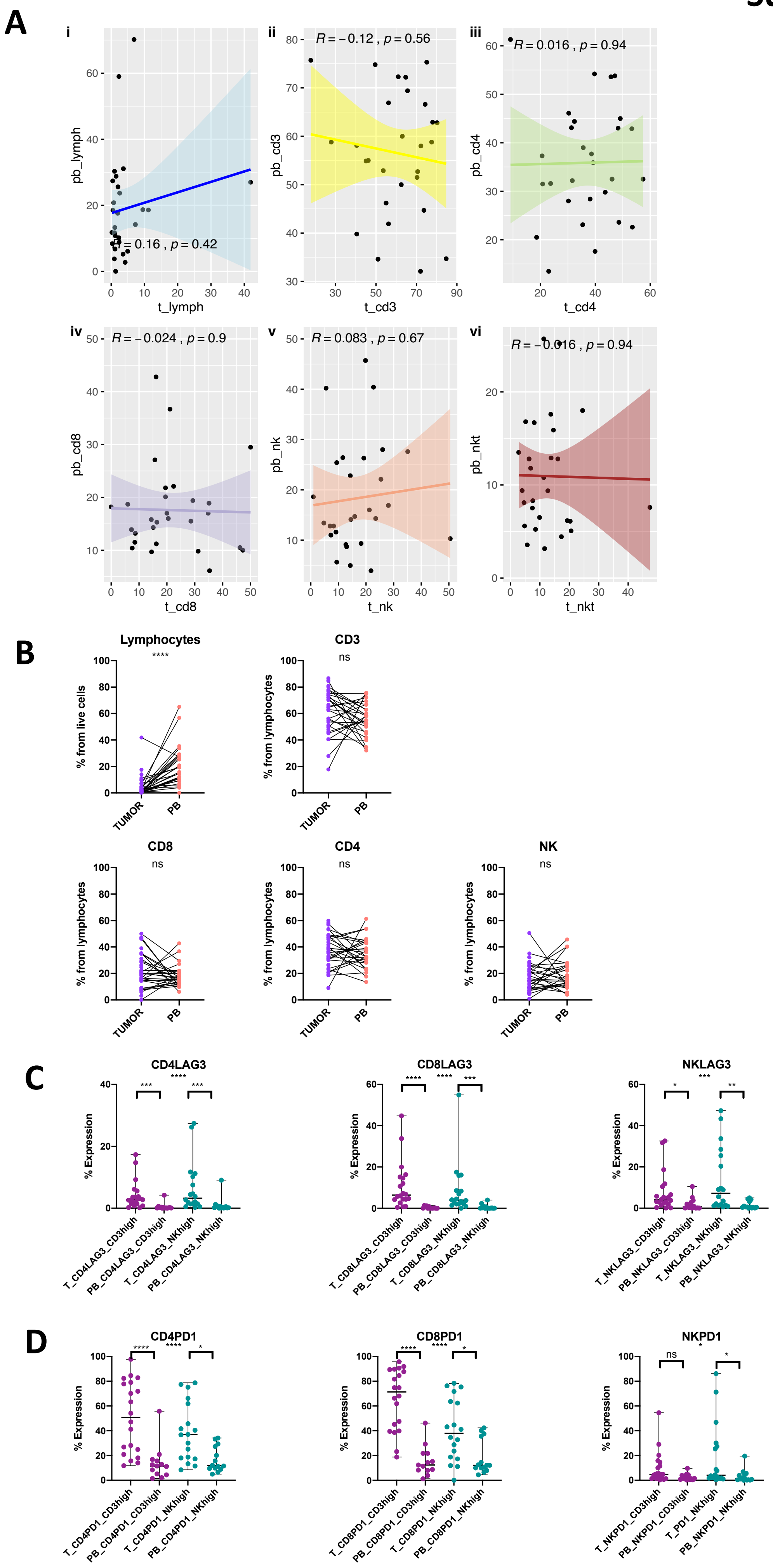

Supplement: Supplemental Material [file KONI_A_1993042_SM1805.zip › supp_all_fin.pdf]
