# Supplementary material for: T and NK cell abundance defines two distinct subgroups of renal cell carcinoma
Source: Oncoimmunology. 2022 Jan 4;11(1):1993042. doi: 10.1080/2162402X.2021.1993042 (PMC8741293; doi:10.1080/2162402X.2021.1993042)
Supplement: Supplemental Material [file KONI_A_1993042_SM1805.zip › supp_tables_fin.zip!/table_s1.pdf]

Supplemental Table S1

Antibody	Fluorochrome	Catalog no	Manufacturer
**CX3CR1**	FITC	341606	BioLegend
**CD16**	PE	561313	BD
**CD3**	PerCP-Cy5.5	332771	BD
**CD4**	PE-Cy7	560649	BD
**TCR γδ**	APC	555718	BD
**CD45**	APC-H7	560178	BD
**CD8**	BV510	563919	BD
**CD56**	BV421	562751	BD
**CD279 (PD-1), clone MIH4**	FITC	557860	BD
**CD223 (LAG-3)**	PE	125209	eBioscience
**CD278 (ICOS)**	PE-Cy7	25-9948-41	eBioscience
**CD152 (CTLA-4)**	APC	560938	BD
**HLA-DR**	BB515	564516	BD
**CD27**	PE	124211	BD
**CD25**	PE-Cy7	101911	BD
**CD11b**	APC	550019	BD
**CD159c (NKG2C)**	AF488	FAB138G	RnD
**CD161**	PE	556081	BD
**CD314 (NKG2D)**	PE-Cy7	562365	BD
**CD159a (NKG2A)**	APC	FAB1059A	RnD
**CD226 (DNAM)**	BB515	565152	BD
**CD57**	PE	560844	BD
**CD335 (NKp46)**	PE-Cy7	562101	BD
**CD337 (NKp30)**	APC	558408	BD
**CD183 (CXCR3)**	AF488	561730	BD
**CCR7**	PE	FAB197P	RnD
**CD45RO**	PE-Cy7	560608	BD
**CD184 (CXCR4)**	APC	560936	BD
**CD274 (PD-L1), clone MIH1**	PE-Cy7	558017	BD
**CD273 (PD-L2), clone MIH18**	APC	557926	BD
