# Supplementary material for: T and NK cell abundance defines two distinct subgroups of renal cell carcinoma
Source: Oncoimmunology. 2022 Jan 4;11(1):1993042. doi: 10.1080/2162402X.2021.1993042 (PMC8741293; doi:10.1080/2162402X.2021.1993042)
Supplement: Supplemental Material [file KONI_A_1993042_SM1805.zip › supp_tables_fin.zip!/table_s2.pdf]

Supplemental Table S2

	Patient ID	Age at surgery	Gender (1=male, 2=female)	Histological type, diagnosis	WHO2016/ISUP	TNM8 (2017)	Tumor diameter (mm)
1	041	74	1	ccrcc	4	pT1b	55
2	056	70	1	ccrcc	3	pT3aN0	135
3	076	71	1	chromophobe rcc	2	pT3a	80
4	078	79	2	ccrcc	3	pT3a	80
5	081	81	2	ccrcc	2	pT1b	60
6	085	71	1	ccrcc	3	pT3a	80
7	096	70	2	ccrcc	3	pT2a	73
8	115	82	1	ccrcc	3	pT2	110
9	125	70	1	ccrcc	2	pT1b	45
10	126	60	2	ccrcc	4	pT2a	71
11	131	80	2	ccrcc	2	pT2a	75
12	141	56	1	oncocytoma	N/A	N/A	70
13	148	85	2	urothelial carcinoma	2	pT1	50
14	159	65	1	ccrcc	2	pT1a	38
15	166	56	1	ccrcc	3	pT3a	80
16	177	70	2	ccrcc	4	pT3aNx	120
17	178	77	2	ccrcc	3	pT3a	55
18	190	69	1	ccrcc	2	pT3aNx	90
19	229	76	1	ccrcc	2	pT3aNx	40
20	255	82	1	ccrcc	3	pT3a	120
21	279	23	1	chromophobe rcc	2	pT2a	100
22	280	85	2	ccrcc	2	pT4	55
23	286	75	2	ccrcc	2	pT2a	85
24	287	68	2	ccrcc	2	pT1b	50
25	292	58	1	ccrcc	3	pT3aNx	60
26	288	57	2	ccrcc	2	pT3a	70
27	289	80	2	ccrcc	4	pT3aNx	100
28	310	53	1	ccrcc	3	pT1b	62
29	346	53	2	ccrcc	2	pT1b	20
30	317	70	1	ccrcc	1	pT1a	60
31	318	59	1	oncocytoma	N/A	N/A	70
32	401	78	2	ccrcc	3	pT2a	70
33	406	72	1	ccrcc	2	pT1b	45
34	413	70	2	ccrcc	2	pT3a	55
35	423	73	2	ccrcc	2	pT2a	80
36	426	85	1	ccrcc and prcc	3	pT3a	36
37	468	73	1	ccrcc	3	pT2a	75
38	476	75	1	ccrcc	2	pT1b	43
39	496	67	1	chromophobe rcc	3	pT3a	90
40	497	56	1	ccrcc	3	pT2a	90
41	499	74	2	papillary RCC	3	pT3a	150
42	500	45	2	ccrcc	3	pT1a	25
43	541	46	2		N/A	N/A	0
44	547	71	2	ccrcc	2	pT2a	80
45	613	67	2	ccrcc	2	pT3a	60
46	616	59	1	ccrcc	2	pT2a	75
47	635	71	1	ccrcc	3	pT1a	25
48	649	69	1	oncocytoma	N/A	N/A	0
49	657	38	2	papillary RCC	2	pT2a	90
50	676	53	1	chromophobe rcc	N/A	pT2b	120
51	680	85	2	oncocytoma	N/A	N/A	0
52	686	60	1	papillary RCC	2	pT1a	40
