# Supplementary material for: T and NK cell abundance defines two distinct subgroups of renal cell carcinoma
Source: Oncoimmunology. 2022 Jan 4;11(1):1993042. doi: 10.1080/2162402X.2021.1993042 (PMC8741293; doi:10.1080/2162402X.2021.1993042)
Supplement: Supplemental Material [file KONI_A_1993042_SM1805.zip › supp_tables_fin.zip!/table_s3.pdf]

Supplemental Table. S3

		Median (%)			Range (%)			p (Kruskal-Wallis)		
	Subsets	T	PB	H	T	PB	H	T vs PB	T vs H	PB vs H
	Lymphocytes	1.91	15.80	0.50	0.17-41.90	0.06-65.10	0.18-19.50	<0.0001	0.2283	<0.0001
	CD3+	64.70	58.00	57.70	17.80-86.80	32.10-75.70	19.10-81-60	0.24	0.2	>0.99
	CD4+	37.30	32.50	34.80	9.08-59.90	13.50-61-30	0.25-54-90	>0.99	>0.99	>0.99
	CD8+	19.50	15.90	15.05	0.00-50.00	6.12-42.80	5.99-37.50	0.53	0.28	>0.99
	NK	14.50	14.70	13.90	0.94-50.50	3.92-45.70	2.39-28.90	>0.99	0.77	0.34
	NKT	8.51	9.40	16.45	1.05-47.40	3.16-25-70	3.30-33.00	>0.99	0.1	0.36
**Markers**										
**LAG3**	CD4LAG3	2.75	0.25	1.63	0.00-39.50	0.02-9.09	0.00-14.50	<0.0001	0.15	0.0022
	CD8LAG3	4.61	0.14	2.55	0.00-78-30	0.00-4.03	0.00-97.70	<0.0001	0.13	<0.0001
	NKLAG3	5.47	0.69	1.60	0.14-50.20	0.00-10.50	0.00-10.50	<0.0001	0.0059	0.06
**PD1**	CD4PD1	41.40	12.25	29.40	8.43-97.70	1.44-55.80	6.56-79.00	<0.0001	0.099	0.0015
	CD8PD1	62.15	14.45	46.20	0.00-97.80	1.27-100.00	6.46-99.10	<0.0001	0.48	0.0004
	NKPD1	6.28	1.52	5.99	0.29-86.10	0.00-20.00	0.44-91.60	<0.0001	>0.99	0.0002
**CTLA4**	CD4CTLA4	0.18	0.04	0.43	0.00-3.15	0.00-95.10	0.00-4.05	0.68	>0.99	0.09
	CD8CTLA4	0.55	0.04	1.08	0.00-4.61	0.00-91.90	0.09-6.89	0.25	0.91	0.0087
	NKCTLA4	0.22	0.04	0.79	0.00-1.57	0.00-17.90	0.00-2.67	0.29	>0.99	0.016
**ICOS**	CD4ICOS	7.96	11.85	3.58	1.39-22.80	2.35-39.50	1.60-20.40	0.71	0.05	0.0001
	CD8ICOS	3.40	2.97	2.62	0.27-16.30	0.12-32.30	0.27-87.20	>0.99	>0.99	>0.99
	NKICOS	0.61	0.04	0.39	0.00-10.80	0.00-7.78	0.00-7.59	0.014	>0.99	0.081
**CXCR3**	CD4CXCR3	5.55	35.65	4.42	0.00-94.70	0.72-63.70	0.00-79.10	0.0001	>0.99	0.0002
	CD8CXCR3	7.44	58.00	5.94	0.00-99.20	0.00-93.80	0.54-98.90	<0.0001	>0.99	<0.0001
	NKCXCR3	9.90	10.50	8.17	0.00-97.40	1.06-38.50	1.15-87.70	>0.99	>0.99	>0.99
**CXCR4**	CD4CXCR4	79.65	85.15	66.60	3.70-100.00	64.00-99.60	21.10-91.80	0.028	0.045	<0.0001
	CD8CXCR4	68.60	80.85	46.90	0.00-98.50	28.20-100.00	17.60-81.90	0.017	0.14	<0.0001
	NKCXCR4	43.40	29.25	54.05	0.00-88.20	4.67-100.00	3.85-85.20	0.90	>0.99	>0.99
**CCR7**	CD4CCR7	22.05	55.40	35.05	3.24-67.80	8.29-87.80	15.10-83.30	0.0057	>0.99	0.12
	CD8CCR7	6.96	16.60	19.00	0.06-40.30	0.00-88.40	1.97-50.30	0.36	0.50	>0.99
	NKCCR7	8.22	0.72	13.45	3.12-22.30	0.06-3.12	1.30-55.10	<0.0001	>0.99	<0.0001
**CD45RO**	CD4CD45RO	94.35	80.25	91.55	0.07-99.30	35.80-97.80	3.34-98.90	0.0018	>0.99	0.038
	CD8CD45RO	85.15	64.60	86.10	0.88-98.70	23.00-97.10	2.97-96.60	0.015	>0.99	0.011
	NKCD45RO	24.80	5.84	17.35	0.22-53.30	0.50-34.10	0.60-41.40	0.0006	>0.99	0.014
**CD57**	CD4CD57	22.45	6.10	13.40	0.00-99.20	0.00-27.70	0.06-42.40	<0.0001	0.025	0.098
	CD8CD57	43.55	34.40	24.50	0.00-78.00	0.00-69.40	0.71-100.00	0.15	0.085	>0.99
	NKCD57	26.90	49.85	51.40	0.10-71.50	0.17-88.40	5.85-91.00	0.0050	0.0058	>0.99
**DNAM**	CD4DNAM	75.40	84.95	63.00	0.64-92.10	28.80-95.70	0.80-91.60	0.0075	>0.99	0.0015
	CD8DNAM	48.10	75.10	48.10	0.22-98.40	13.00-96.60	0.08-100.00	<0.0001	>0.99	<0.0001
	NKDNAM	30.15	80.30	30.40	0.35-93.90	3.57-97.90	0.00-79.30	<0.0001	>0.99	<0.0001
**NKG2A**	CD4NKG2A	1.41	0.70	1.27	0.14-94.70	0.09-16.90	0.00-56.60	0.015	>0.99	0.14
	CD8NKG2A	2.42	0.61	2.64	0.00-88.20	0.05-37.20	0.87-11.00	0.001	>0.99	0.0077
	NKNKG2A	11.05	5.14	4.80	0.38-47.60	0.34-18.00	0.39-45.50	0.0017	0.0025	>0.99
**NKG2D**	CD4NKG2D	6.12	1.70	5.19	0.49-98.50	0.22-15.90	0.00-36.20	<0.0001	>0.99	<0.0001
	CD8NKG2D	49.70	32.00	73.05	8.12-93.50	2.19-84.80	26.60-84.00	0.35	0.13	0.0079
	NKNKG2D	30.25	27.10	17.35	2.67-81.30	1.10-79.10	0.53-72.90	0.6	0.057	0.83
**CD161**	CD4CD161	5.76	19.10	3.98	0.15-29.40	1.26-32.60	0.80-52.30	0.02	>0.99	0.0004
	CD8CD161	3.33	7.14	3.78	0.82-43.10	0.64-92.90	0.21-35.20	0.19	>0.99	0.076
	NKCD161	21.40	74.55	5.35	0.98-42.70	4.11-92.40	1.01-49.20	<0.0001	>0.99	<0.0001
**NKG2C**	CD4NKG2C	5.96	0.37	4.17	0.38-48.60	0.04-2.24	0.16-17.40	<0.0001	>0.99	<0.0001
	CD8NKG2C	10.15	0.97	3.87	0.71-63.10	0.00-39.20	0.38-12.10	0.0001	0.53	0.038
	NKNKG2C	29.40	11.05	16.50	9.37-83.60	2.28-68.20	3.33-59.90	0.008	0.033	>0.99
**NKp30**	CD4NKp30	0.42	0.15	26.10	0.00-68.80	0.00-50.80	1.36-58.60	0.54	0.0042	<0.0001
	CD8NKp30	1.47	1.39	15.60	0.15-26.20	0.00-99.20	0.00-44.70	>0.99	0.012	0.0029
	NKNKp30	31.40	78.30	6.13	1.89-64.50	1.49-98.10	0.50-44.30	0.0034	0.40	<0.0001
**NKp46**	CD4NKp46	3.31	0.49	19.50	0.22-13.10	0.00-8.40	0.69-52-50	0.042	0.093	<0.0001
	CD8NKp46	7.85	0.52	24.10	0.35-76.20	0.00-17.60	2.08-59.80	0.0022	0.35	<0.0001
	NKNKp46	35.05	72.20	23.80	3.29-58.10	9.01-85.80	10.60-64.50	0.0002	>0.99	0.0001
**CX3CR1**	CD4CX3CR1	5.69	4.99	5.14	0.11-29.00	0.29-49.10	0.26-11.40	>0.99	>0.99	>0.99
	CD8CX3CR1	7.63	44.85	3.88	0.83-21.60	5.44-81.70	0.26-29.30	0.0001	0.77	<0.0001
	NKCX3CR1	37.10	93.95	22.25	4.91-88.80	38.60-100.00	6.34-86.60	<0.0001	>0.99	<0.0001
**CD11b**	CD4CD11b	4.68	1.50	5.03	1.09-27.30	0.00-13.60	0.06-15.20	0.0033	>0.99	0.086
	CD8CD11b	9.44	9.60	3.37	1.86-41.60	0.21-99.30	0.71-20.30	>0.99	0.51	0.81
	NKCD11b	59.15	78.15	62.60	30.80-84.00	25.40-98.10	24.10-91.00	0.072	>0.99	0.15
**CD25**	CD4CD25	3.13	43.85	7.52	1.01-11.50	9.20-99.30	0.80-27.90	<0.0001	>0.99	<0.0001
	CD8CD25	1.63	10.55	3.45	0.17-5.99	0.65-100.00	0.08-15.30	0.0001	0.75	0.029
	NKCD25	4.70	1.39	3.81	0.21-15.00	0.22-66.40	0.00-11.60	0.43	>0.99	0.37
**CD27**	CD4CD27	3.31	89.05	21.20	0.10-17.90	56.00-97.00	8.58-52.50	<0.0001	0.26	<0.0001
	CD8CD27	4.50	62.10	18.50	0.28-20.90	34.80-96.00	6.46-44.70	<0.0001	0.26	<0.0001
	NKCD27	1.51	4.92	3.54	0.26-6.11	0.78-18.20	0.28-15.60	0.0003	0.029	>0.99
**HLADR**	CD4HLADR	55.00	9.27	42.10	24.00-91.00	2.62-22.80	19.30-63.20	<0.0001	0.92	<0.0001
	CD8HLADR	59.15	16.45	37.90	42.70-96.20	1.95-79.50	16.50-74.20	<0.0001	0.18	0.0043
	NKHLADR	48.65	6.90	38.60	12.80-86.50	1.77-25.50	12.10-90.90	<0.0001	>0.99	<0.0001
